# Integrated Algal Bioenergy Platforms: Advances in Microbial Fuel Cell Integration and Nanotechnology-Enabled Biorefineries

**DOI:** 10.3390/nano16181182

**Published:** 2026-09-18

**Authors:** Yehia S. Mohamed, Sinclair Steele, Doaa S. R. Khafaga, Youssef Basem, Arwa M. Shawky, Youssef G. Mostafa, Samar M. Solyman

**Affiliations:** 1 Department of Pathological Sciences, College of Medicine, Ajman University, Ajman P.O. Box 346, United Arab Emirates; 2 Department of Microbiology and Immunology, Faculty of Pharmacy (Boys), Al-Azhar University, Cairo 11765, Egypt; 3 Department of Basic Medical Sciences, Faculty of Medicine, Galala University, New Galala City, Suez 43511, Egypt; 4 Medical and Pharmaceutical Industrial Biotechnology Department, College of Biotechnology, Misr University for Science and Technology (MUST), 6th of October City, Giza 12566, Egypt; 200025117@must.edu.eg; 5 Department of Microbiology and Immunology, Faculty of Pharmacy, Sinai University-Kantara Branch, Ismailia 41522, Egypt; a.ahmed2392@su.edu.eg (A.M.S.); samar.mansour@su.edu.eg (S.M.S.); 6 Department of Microbiology and Immunology, Faculty of Pharmacy, Suez Canal University, Ismailia 41522, Egypt

**Keywords:** microalgae, microbial fuel cells, MFC, biodiesel, bioethanol, biogas, biohydrogen

## Abstract

The use of algae-based bioenergy platforms in establishing circular bioeconomy systems is due to their capacity to generate biomass of renewable origin, using solar energy and carbon dioxide (CO_2_), while the process also involves remediation and recovery of resources. The introduction of algae to bioenergy systems is still limited by obstacles related to biomass productivity, efficiency of harvesting, and energy demands of further utilization of biomass, not to mention lack of system integration. The present paper provides a review of the new developments in the area of algal bioenergy platforms, focusing on the use of algae-assisted microbial fuel cells (MFCs) and biotech solutions based on nanotechnology. The combination of algae with MFC technology allows for the generation of bioelectricity and the use of algal systems for carbon fixation, nutrient removal, bioconversion of biomass, etc. Meanwhile, nanotechnology has become an important approach to enhance the development of algal bioenergy technologies by providing better cultivation activity, light absorption, nutrient transport, biomass extraction, catalysis, and electrodes. Advanced nanomaterials, such as magnetic nanoparticles, metal oxides, carbon-containing nanostructures, and combined nanomaterials, have allowed the industry to overcome the most important problems in algal biomass processing and bioelectrochemical energy production. At the same time, integrated algal biorefineries make it possible to transform a variety of biomass forms into biofuels or something even more valuable. Nevertheless, despite achieving significant resolution of many concerns, the scalability of technologies and the safety of the nanoparticles are still among major challenges to industrial implementation. The further development of algae-based technologies should allow sufficient integration of advanced nanomaterials, genetically engineered microbes, and scaling of reactor technologies. This review provides a unique integrated framework connecting algae cultivation, microbial fuel cell technologies, and nanotechnology-enabled biorefineries toward scalable circular bioenergy systems.

## 1. Introduction

As fossil fuels are being quickly used up, and with large amounts of greenhouse gases (GHGs) being created this has driven the world to find sustainable, renewable, and green energy alternatives [1]. While fossil fuels are still being used for most of the world’s energy, the amount of fossil fuels being used has caused a great deal of climate change, environmental issues, and energy insecurities. With the need to become carbon-neutral, and to meet global climate targets, much research has gone into finding renewable biofuels that can replace traditional petroleum fuels [2]. Of all the different types of renewable feedstocks that have been looked at, microalgae and macroalgae have emerged as some of the best candidates for next-generation biofuels because of their great growth rates, and their ability to fix carbon, as well as their versatility in converting the biomass into usable fuels [3].

Algae have been designated as 3rd generation biofuel feedstocks, whilst 1st and 2nd generation biofuels are derived from edible crops and lignocellulosic biomass, respectively. Algal cultivation does not compete with food production, nor is it necessary to produce algae on arable land. Algae can be cultivated in a variety of environments, including saline water, industrial by-products, and municipal sewage. In addition, algae have photosynthetic efficiencies far greater than terrestrial plants and can store large amounts of lipids, carbohydrates, and proteins that can be derived to produce a multitude of biofuels. As a result, algae play an important role in the development of future energy systems that are sustainable and in initiatives to develop a circular bioeconomy [4].

Recent advances in algal biotechnology have led to a wider variety of biofuels that can be produced from algal biomass. Depending on their species and method of processing, algae will produce various forms of biodiesel, bioethanol, biogas, biohydrogen, biochar, and renewable substitutes for fossil fuels. Notably, a large market is developing for the use of algal oil as biodiesel, as it has a high oil content and rapid rates of biomass production. Nevertheless, significant obstacles to large-scale commercialization exist, mainly associated with algal cultivation harvesting, de-watering of biomass, and downstream processing costs [1,4].

Algae not only produces energy but helps the environment because they absorb carbon dioxide and can be used to treat wastewater. Wastewater is rich in nutrients, which makes it a low-cost option for growing algae, while at the same time lowering nitrogen and phosphorus levels in the water before it is released back into the environment. The use of algae and wastewater in this way can help create resources, control pollution, and produce biomass, which allows producing algae for biofuel to be more sustainable and economically viable. The use of wastewater to grow algae is considered a new way to reduce the operational costs and improve the environmental performance of producing algae for biofuel [5,6].

Green hydrogen production has emerged as an important pathway for sustainable energy generation, with current approaches including water electrolysis technologies, such as alkaline, proton exchange membrane, and solid oxide electrolysis, as well as biological routes based on microbial and algal systems. Although electrochemical water splitting provides high-purity hydrogen, its sustainability depends strongly on renewable electricity availability and material requirements. Biological hydrogen production using microalgae offers an alternative approach by coupling solar energy conversion with carbon fixation and hydrogen generation through photosynthetic pathways. However, improving hydrogen productivity and overcoming limitations associated with oxygen sensitivity, metabolic regulation, and reactor scalability remain essential challenges for practical implementation [7].

Research is also being conducted on employing algal species in MFCs. MFCs are bio-electrochemical systems that convert chemical energy stored in organic matter directly into electricity by way of microorganisms. MFCs typically require oxygen to operate, which must come from an outside source for the reaction to occur at the cathode of the MFC. Because algae can produce oxygen through photosynthesis, this allows for better performance at the cathode and decreases the amount of electricity required to accomplish the reaction. Algae assist MFCs in producing bioelectricity while removing contaminants from wastewater as well as absorbing CO_2_ and producing biomass. Research has shown that algal species can be used as biocathodes to improve the performance and efficiency of MFCs. Researchers have identified that the incorporation of algal biocathodes and windows into MFCs resulted in significant increases in both power output and efficiency of treatment [8,9].

Algal bioenergy production has been improved using nanotechnology as a transformative tool, providing new ways to enhance efficiency in areas like light transfer, nutrient transportation, harvesting of biomass, lipid extraction, and efficiency of converting biofuels. Nanomaterials can increase the speed at which transesterification reactions take place, as well as increase electron transfer opportunities in microbial fuel cells and therefore lead to more efficient biodiesel creation [10]. As algal biorefineries continue to gain attention as a means of maximizing biomass utilization and improving economics, recent studies have shown that the biofuels-only focus of an algal refinery is changing to an integrated means of producing multiple value-added products, including biofuels, pigments, nutraceuticals, pharmaceuticals, bioplastics, fertilizers, and animal feed. This integrated system closely follows the principles of a circular bioeconomy by minimizing waste and maximizing the efficiency of resources [10].

While there has been substantial progress made in research of algal bioenergy technologies there are still many challenges associated with the production of algal biofuels such as the cost to cultivate them, the ability to harvest them, the ability to process them once harvested down to usable fuels, the scalability of these systems, and their long-term sustainability as viable economical sources of energy. In addition, while many researchers have independently published reviews regarding algal biofuels in general, microbial fuel cells produced from algal material, and the application of algal biomass to treat wastewater, and have used nanotechnology to enhance the processes and systems associated with using algal biomass to generate fuel from algae, there have not been reviews published that synthesize all current developments and provide insight into the interconnected nature of these research areas. Therefore, a comprehensive synthesis of the recent advancements that have been made is required to identify where knowledge gaps currently exist and future opportunities for developing technologies related to algal biofuels [1].

The future development of third-generation algal biofuels is increasingly influenced by emerging regulatory frameworks and industrial demands for low-carbon energy alternatives. In particular, the aviation sector has identified sustainable aviation fuels (SAFs) as a critical pathway for reducing greenhouse gas emissions from hard-to-decarbonize transportation sectors. Due to their high lipid productivity, rapid biomass generation, and ability to utilize non-arable land and alternative carbon sources, microalgae have been investigated as potential feedstocks for SAF production. Similarly, renewable diesel mandates and low-carbon fuel policies have increased interest in lipid-derived fuels that can serve as drop-in replacements for conventional diesel. However, achieving commercial viability requires alignment between technological development, regulatory incentives, and market requirements, in addition to improvements in cultivation efficiency, conversion processes, and overall economic performance [11].

Unlike previous reviews that have primarily examined algal biofuels, microbial fuel cells, or nanomaterial applications as separate research areas, this review provides an integrated perspective on algae-based bioenergy platforms by analyzing the synergistic interactions among algal biomass production, bioelectrochemical energy recovery, and nanotechnology-driven process optimization. The unique contribution of this review lies in evaluating how these interconnected strategies can advance circular biorefineries while addressing key challenges related to scalability, sustainability, and industrial translation.

This review will provide an overview of algal bioenergy production pathways including but not limited to biodiesel, bioethanol, biogas, and biohydrogen; highlight recent advancements in using algae to enhance microbial fuel cells; and review the use of nanotechnology.

## 2. Methodology

A structured narrative review approach was adopted to synthesize recent advances in integrated algal bioenergy platforms, with particular emphasis on algae-integrated MFC systems and nanotechnology-enabled algal biorefineries. A narrative methodology was selected because the objective of this review was to provide a multidisciplinary and conceptual synthesis of interconnected technologies rather than perform a quantitative meta-analysis of homogeneous datasets. This approach enables the integration of diverse research areas, including algal cultivation, bioelectrochemical energy recovery, nanomaterial-assisted process optimization, and circular biorefinery strategies.

Literature was identified through searches of major scientific databases, including PubMed, Scopus, Web of Science, and Google Scholar. The search covered peer-reviewed publications from 2020 to 2026, with selected earlier studies included when required to provide fundamental background and historical context. The search strategy employed combinations of keywords and Boolean operators related to “microalgae bioenergy,” “algal biorefinery,” “microbial fuel cells,” “photosynthetic bioelectrochemical systems,” “nanotechnology,” “nanomaterials,” “biofuel conversion,” “wastewater treatment,” and “circular bioeconomy.”

Studies were included based on their relevance to integrated algae-based bioenergy systems, including research addressing (i) algal biomass production and valorization, (ii) algae-assisted MFC configurations and bioelectrochemical applications, (iii) nanotechnology-based approaches for improving cultivation, harvesting, catalytic conversion, and electrode performance, and (iv) integrated biorefinery concepts involving energy recovery and resource utilization. Peer-reviewed original research articles and high-quality review articles providing substantial mechanistic or technological insights were prioritized.

Studies were excluded if they were non-English publications, conference abstracts, opinion articles, or lacked direct relevance to algae-based energy systems, MFC integration, nanotechnology applications, or sustainable biorefinery approaches. Titles and abstracts were initially screened to identify potentially relevant studies, followed by full-text evaluation based on predefined relevance criteria. The selected literature was then organized into thematic categories focusing on algae-integrated MFC platforms and nanotechnology-enabled algal bioenergy systems.

**Figure 1:**
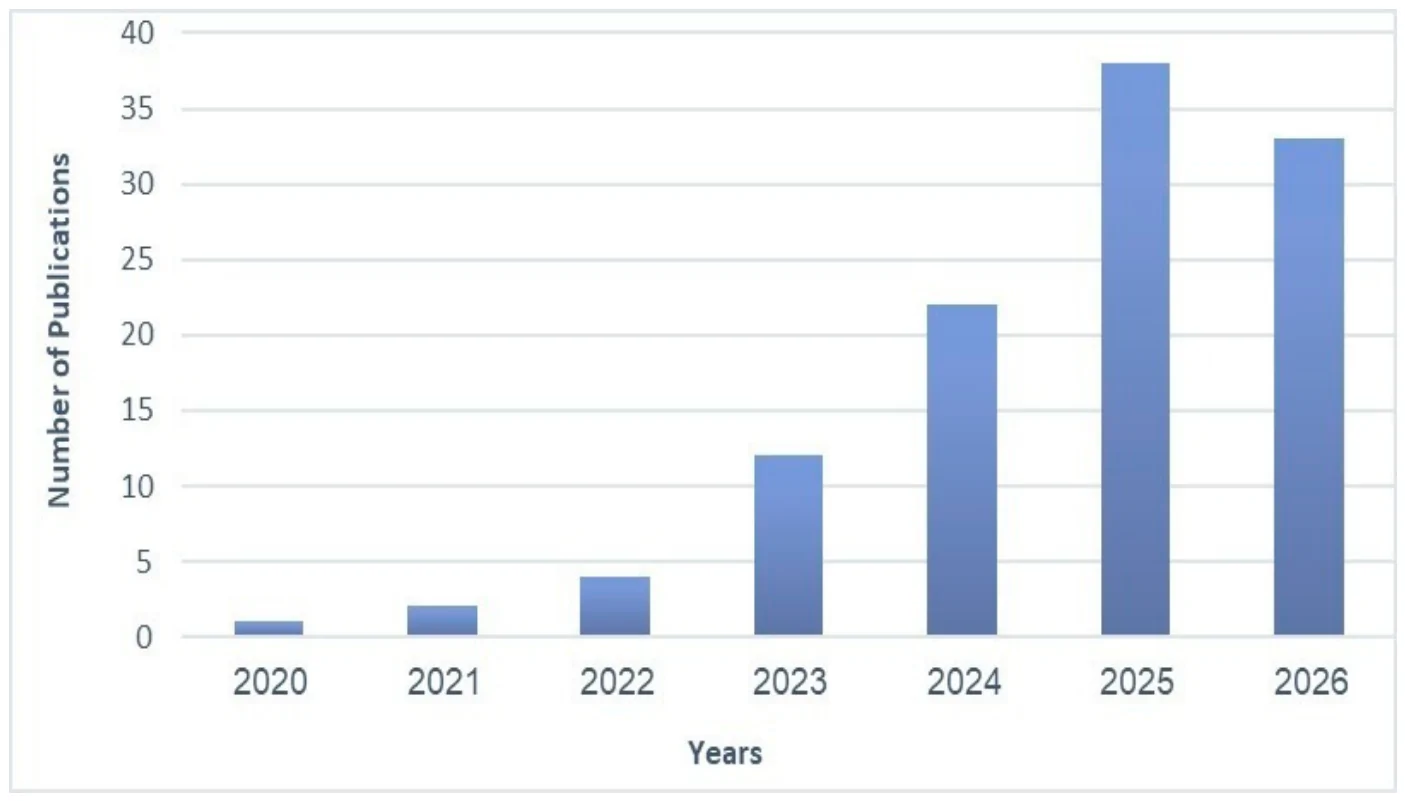
Annual publication trends (2020–2026) illustrating research growth in algae-based bioenergy, microbial fuel cells, and nanotechnology-enhanced bioenergy systems. The 2026 value represents publications retrieved up to the date of literature search and does not indicate a complete annual count.

Because this review follows a narrative synthesis framework, a formal risk-of-bias assessment or quantitative quality scoring was not performed. Instead, emphasis was placed on studies with clear experimental methodologies, relevant performance evaluation parameters, and reproducible findings. The extracted information included algal species, cultivation strategy, biomass conversion pathway, MFC configuration, nanomaterial type and function, performance improvements, and major technological limitations. The collected evidence was critically analyzed to identify current advances, remaining challenges, and future opportunities for developing scalable and sustainable algal bioenergy platforms. A qualitative comparative synthesis was performed. Studies were categorized into two major thematic domains: algae-integrated microbial fuel cell platforms and nanotechnology-enabled algal biorefineries, with supporting evidence from cultivation, biomass conversion, and circular resource recovery studies. Emphasis was placed on identifying cross-system integration strategies and scalability potential. Although methodological heterogeneity limited direct quantitative comparison, this approach enabled a focused and high-resolution synthesis of current evidence, highlighting key technological trends, limitations, and future directions in integrated algal bioenergy systems, as illustrated in Figure 1.

## 3. Nanotechnology-Enabled Algal Bioenergy Systems

### 3.1. Biological Characteristics and Advantages

Microalgae are tiny microscopically small but very productive organisms that carry out photosynthesis using sunlight and converting CO_2_ to produce biomass that is rich in lipids, carbohydrates, proteins, and other high-value products. Due to their rapid growth rates, high efficiency of photosynthesis, and ability to accumulate large amounts of energy-rich material, they represent excellent feedstocks for renewable biofuels. For example, compared to land-based energy crops, microalgae produce much higher rates of biomass per unit area and use far less land and freshwater, thus providing a suitable feedstock for sustainable bioenergy at large scales [12]. Marine algae microalgae and macroalgae are increasingly recognized as potential feedstocks for future biofuels because of their abundance, carbon sequestration characteristics, and ability to be grown in saline environments not competing with agricultural land. The cultivation of marine algae for biofuels helps with carbon capture and reduces the pressure on freshwater sources as we move towards a circular and low-carbon bioeconomy [13]. Microalgae not only have the potential to produce renewable fuels; they also represent a valuable biorefinery that can produce many different products, such as bioactive compounds, pharmaceuticals, nutraceuticals, animal feed, fertilizers, and industrial chemicals. Microalgae can also harness industrial flue gases and wastewater streams during their growth processes, thus adding to the overall environmental and economic sustainability of these organisms through the integration of biomass production and pollution reduction/environmental remediation [12,13].

Strain selection represents a critical determinant in the design of efficient algal bioenergy systems, as different algal species exhibit distinct physiological characteristics that influence cultivation requirements and downstream processing strategies. Lipid-rich strains, such as *Nannochloropsis* and *Chlorella* species, are particularly suitable for biodiesel production and require cultivation conditions that promote lipid accumulation followed by efficient harvesting and lipid extraction processes. In contrast, carbohydrate-rich strains may be more appropriate for bioethanol production due to their higher fermentable sugar content and compatibility with hydrolysis and fermentation processes. Furthermore, robust strains with high growth rates and environmental tolerance are preferred for large-scale open pond cultivation and wastewater-based systems, whereas more sensitive high-value strains may require controlled photobioreactor conditions. Therefore, matching algal strain characteristics with appropriate cultivation systems and downstream conversion pathways is essential for improving the productivity, economic feasibility, and scalability of algal bioenergy platforms [14].

### 3.2. Cultivation Systems

Any type of cultivation system will affect the amount of biomass produced from different types of algae, how much it costs to operate, how susceptible it is to being contaminated, and ultimately how much biofuel is produced. Algal cultivation technologies are typically grouped into those that use open systems raceway ponds and those that use closed systems, including many types of photobioreactor (PBR) configurations. Open ponds have lower capital and operating costs than closed systems; however, they are also subjected to contamination and evaporation losses and generally have poor control of environmental conditions. Closed PBRs provide greater control of sunlight, temperature, pH, nutrient supply, and delivery of CO_2_, which results in higher rates of biomass production and more stable cultures [15].

Recent developments in PBR engineering have concentrated on improving the hydrodynamic, light distribution, and mass transfer properties of PBRs. Innovative airlift and flat panel PBRs have been developed that demonstrate significant improvements over conventional systems in terms of mixing efficiency, CO_2_ utilization, and amount of nutrients removed from solutions, resulting in increased biomass production and lower cultivation costs. It is anticipated that these types of technologies will improve the ability to scale and commercially implement algal biofuel production systems [16].

**Figure 2:**
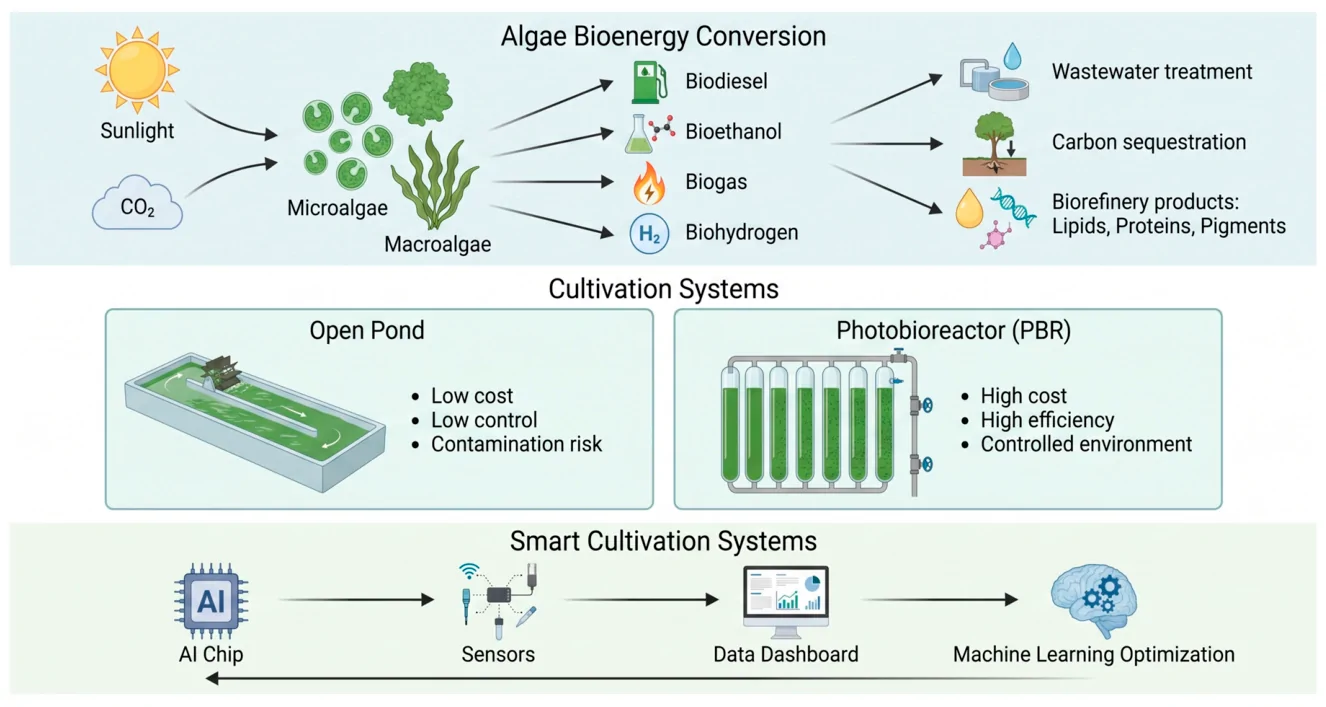
Schematic representation of algae as a bioenergy feedstock and its major cultivation systems.

The development of algae has various cultivation parameters, such as light intensity, photoperiod, CO_2_ concentration, temperature, nutrient availability, and hydraulic retention time, that will have a large impact on biomass productivity and biochemical composition. The optimization of these parameters will lead to increased lipid accumulation, increased carbon fixation rates, and increased nutrient removal efficiency. The literature has shown that mixed microalgal consortia typically outperform monocultures about biomass productivity, environmental resiliency, and wastewater treatment efficiency according to recent pilot-scale intermixed studies [17]. To overcome the cost limitations associated with closed cultivation systems, researchers have been using advanced raceway pond designs and integrating strategies for recycling nutrients and using wastewater as a source for the growth of algae. It is well-documented that high-rate raceway ponds and nutrient recovery technologies significantly reduce the use of freshwater and develop comparable biomass productivity while enhancing the sustainability of large-scale algal cultivation operations [18]. Open pond systems remain the most economically attractive option for large-scale algal cultivation due to their lower capital and operational costs and simpler construction requirements. However, their performance is strongly influenced by environmental fluctuations, including temperature variation, evaporation, light limitation, and higher contamination risks, which can reduce biomass productivity and culture stability. In contrast, closed PBR systems provide superior control over cultivation parameters such as light intensity, temperature, pH, nutrient availability, and CO_2_ supply, resulting in higher biomass productivity and improved culture consistency. Nevertheless, PBRs require higher initial investment, greater energy input for mixing and operation, and more complex maintenance procedures, which can limit economic scalability. Therefore, open ponds are generally more suitable for low-cost, large-scale biomass production, whereas PBRs are advantageous when higher productivity, controlled cultivation conditions, and production of high-value algal products are prioritized. Hybrid approaches combining the scalability of open systems with the control advantages of PBRs may represent a promising strategy for future industrial algal biorefineries [19]. In addition, there has been an increased incorporation of digital technologies such as artificial intelligence, machine learning, and sensor-based monitoring systems into algal cultivation platforms. These smart cultivation systems optimize growth conditions in real time, enable predictive biomass modelling, and provide automated control of the cultivation process, resulting in increased productivity and lower operating costs at commercial-scale facilities [19]. As illustrated in Figure 2, algae-based bioenergy systems integrate biological productivity, environmental remediation, and process optimization. Microalgae and macroalgae can convert sunlight and CO_2_ into biomass suitable for multiple biofuel pathways, while cultivation performance depends strongly on the selected system, ranging from low-cost open ponds to highly controlled photobioreactors supported by smart monitoring technologies.

## 4. CO_2_ Sequestration and Environmental Benefits

One of the key benefits to the environment that microalgae offer is that they have been shown to be capable of sequestering atmospheric and industrial CO_2_ at the same time as providing biomass that can be used to produce biofuels. When compared to terrestrial plant systems, microalgae can sequester CO_2_ at a much greater rate due to their much greater photosynthetic and carbon-fixing efficiencies. Microalgae can also survive and thrive under relatively high levels of CO_2_ that would normally be present in industrial flue gas emissions. Because of these advantages, microalgae are being investigated as potential candidates for incorporation into biological carbon capture and utilization (BCCU) systems [20,21].

Recent research has suggested that integrating the cultivation of microalgae with wastewater treatment systems to create systems that could allow for CO_2_ sequestration, removal of nutrients from wastewater, and the production of biomass from microalgae may be an efficient approach to address environmental issues. This type of system will utilize wastewater that is high in nitrogen and phosphorus as nutrient sources and remove CO_2_ from industrial emissions as part of a circular and resource-efficient process to remediate the environment. Bacteria–microalgae consortia provide a particularly promising avenue for research as these systems have demonstrated improved carbon utilization efficiency, wastewater treatment effects, and overall biomass yield because of the synergistic interactions between the two types of microorganisms [22,23].

Although algae-based systems provide significant CO_2_ capture benefits during cultivation, it is important to recognize that carbon fixation associated with biofuel production does not represent permanent carbon sequestration. When algal biomass is converted into fuels and subsequently combusted, a substantial fraction of the captured carbon is released back into the atmosphere, resulting in a short-term carbon cycle rather than permanent carbon storage. Therefore, the environmental advantage of algal bioenergy depends on achieving a favorable net carbon balance through reduced fossil carbon inputs, efficient cultivation strategies, utilization of waste-derived nutrients and CO_2_ sources, and optimization of downstream processing energy requirements. Life-cycle assessment approaches are essential for accurately evaluating the true climate benefits of algae-based bioenergy systems [24].

Algal-based wastewater treatment systems provide an additional environmental benefit through efficient recovery of nitrogen and phosphorus nutrients while simultaneously generating valuable biomass. Reported nutrient removal efficiencies vary considerably depending on algal species, wastewater composition, and cultivation conditions, with many studies demonstrating substantial reductions in total nitrogen and phosphorus concentrations. However, the suitability of wastewater-grown algal biomass for biofuel production requires careful evaluation. Although nutrient-rich wastewater can reduce cultivation costs and support biomass generation, the recovered biomass may exhibit variable biochemical composition and potential accumulation of contaminants, including heavy metals and organic pollutants. Therefore, wastewater-derived algal biomass may be more suitable for integrated biorefinery approaches, where lipid-rich fractions can be directed toward biodiesel production while residual biomass can be utilized for anaerobic digestion or other conversion pathways. Comprehensive assessment of biomass quality and downstream processing requirements is essential to maximize both environmental and energy benefits [25]. The comparative CO_2_ fixation performance of different algal species under various cultivation conditions is summarized in Table 1, highlighting the effects of algal strain selection, cultivation systems, and carbon availability on carbon sequestration efficiency.

## 5. Biodiesel Production from Algae

To produce biodiesel from microalgae, intracellular lipids must first accumulate inside the cell before being extracted and converted into biodiesel. Nitrogen limitation, concentration of CO_2_ and light intensity are some of the cultural stresses that can have a significant impact on the rate of lipid accumulation because they can affect how the organism produces energy to synthesize neutral lipids. The bottleneck here is that increasing lipid production usually comes at the expense of biomass production and vice versa; therefore, both are key parameters affecting the efficiency of biodiesel production [31].

Improvements in lipid recovery from microalgae can result from the advancement of extraction techniques. Many traditional methods of lipid extraction like Soxhlet or blight and dyer extraction have started to be phased out or have been complemented by newer extraction methods, including microwave-assisted extraction, ultrasound-assisted extraction, supercritical CO_2_ extraction, and ionic liquids. These newer extraction methods provide higher extraction efficiency of lipids, lower toxicity of solvents used during extraction, and higher yields of lipids obtained during extraction.

To overcome the trade-off between biomass productivity and lipid accumulation, advanced cultivation strategies have focused on separating biomass growth and lipid induction phases. Two-stage cultivation systems allow algae to first grow under nutrient-sufficient conditions to maximize biomass production, followed by controlled stress conditions, such as nitrogen limitation or nutrient deprivation, to stimulate intracellular lipid accumulation. This approach improves overall lipid productivity compared with continuous stress exposure, which may reduce cell growth and biomass yield. Additional strategies, including mixotrophic cultivation, optimized stress duration, and selection of high-performing strains, have also been investigated to enhance lipid production while maintaining efficient biomass generation. Therefore, integrating growth optimization with targeted lipid induction represents a promising strategy for improving the economic feasibility of algal biodiesel production [32].

**Figure 3:**
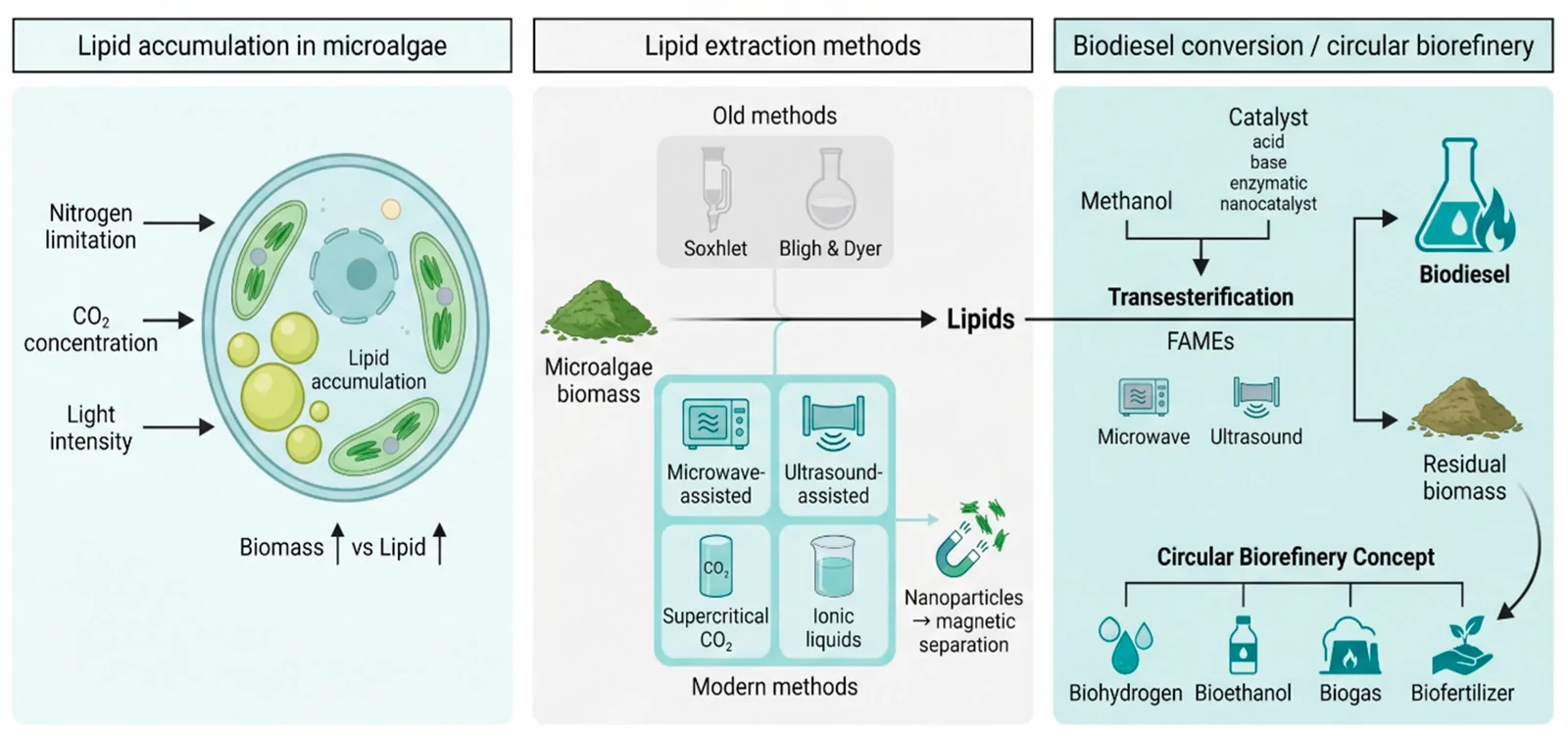
Schematic representation of lipid accumulation, biomass harvesting, lipid extraction, and biodiesel production from microalgae within a circular biorefinery framework. The figure illustrates microalgal cultivation, stress-induced lipid enrichment, conventional and advanced extraction approaches, transesterification of lipids into biodiesel, and valorization of residual biomass into value-added products, emphasizing resource recycling and sustainable bioeconomy principles.

Additionally, there are emerging separation harvesting approaches using nanotechnology that have demonstrated high efficiencies for both lipid extraction and biomass recovery. An example of this would be the use of nanoparticles to facilitate magnetic separation of biomass and lipids in a single operation, thus reducing downstream processing costs and increasing the capacity at which microalgae can be produced [33]. Finally, with the increased commercial/industrial interest in these types of technologies, integrated biorefinery approaches are becoming increasingly popular. In these processes, the extraction of lipids along with the recovery of other valuable co-products like pigments, proteins, and polysaccharides will help to maximize the overall economic viability and sustainability of algal biodiesel production systems [34]. As illustrated in Figure 3, microalgal biodiesel production involves a multi-step process starting from lipid accumulation under stress conditions such as nitrogen limitation, elevated CO_2_ concentration, and controlled light intensity.

### 5.1. Transesterification

Biodiesel is made from microalgal lipids and typically requires converting them into fatty acid methyl ester (FAME) through transesterification, which mixes the lipids with short-chain alcohol (e.g., methanol) and a catalyst, e.g., acid, base, enzymatic, or heterogeneous. The efficiency of the transesterification reaction depends on the catalyst type, temperature, oil-to-alcohol ratio, and moisture content. Recent research has demonstrated the benefits of in situ transesterification, where lipids are extracted and converted at the same time by reducing the amount of solvent needed to create a higher yield overall. In-situ transesterification provides a beneficial process for wet microalgal biomass when compared to previous methods, due to the reduced complexity in processing and energy requirements [31].

Nanocatalysts have been shown to be highly effective in the production of biodiesel, particularly with metal oxide-based and magnetic nanoparticles as they demonstrate a high surface area and are reusable; additionally, they provide improved reaction kinetics versus conventional catalysts. Utilizing microwave-assisted and ultrasound-assisted methods for transesterification provides additional methods for the industry to improve the overall yields in procedural time. Lastly, the residual biomass produced after the extraction of lipids may be utilized for biohydrogen, bioethanol, biogas, or biofertilizer, which supports a circular biorefinery approach that increases resource utilization and promotes sustainability throughout the entire supply chain of the process [35,36].

### 5.2. Comparative Biochemical and Thermochemical Conversion Pathways: Hydrothermal Liquefaction

In addition to biochemical conversion routes, thermochemical approaches, particularly hydrothermal liquefaction (HTL), have gained increasing attention as an alternative pathway for algal biofuel production. Unlike conventional lipid extraction and transesterification processes, HTL can directly process wet algal biomass under elevated temperature and pressure conditions, eliminating the energy-intensive drying step required for lipid recovery. During HTL, the entire algal biomass, including lipids, proteins, and carbohydrates, is converted into a bio-crude product that can be further upgraded into transportation fuels. From a techno-economic perspective, HTL may provide advantages through improved energy efficiency and higher resource utilization; however, its feasibility depends on factors such as biomass cultivation costs, conversion efficiency, reactor requirements, and downstream upgrading demands. Key performance indicators, including energy return on investment (EROI) and minimum fuel selling price (MFSP), are critical for evaluating HTL competitiveness. Studies have shown that MFSP is strongly influenced by feedstock cost, biomass productivity, and conversion yield, while improving process integration and valorization of co-products can enhance the overall economic viability of algal HTL pathways [37].

## 6. Bioethanol Production from Algal Carbohydrates

Microalgae are a promising source of feedstocks for the sustainable production of bioethanol because they contain large amounts of carbohydrates, especially glucose-containing polysaccharides. Microalgae grow rapidly and can grow photosynthetically efficiently, accumulating large portions of carbohydrates in response to stress conditions, i.e., they stress out when exposed to light. They are excellent candidates to provide renewable fuels because they offer advantages over lignocellulosic biomass. For example, the cultivation cycle for microalgae is shorter than for lignocellulosic biomass, and microalgae do not contain lignin, making it easier to process and hydrolyze microalgae after they have been harvested [38].

Recent advances in the optimization of metabolism and culture conditions show that nutrient limitation, specifically nitrogen starvation, can greatly increase the amount of intracellular carbohydrates, as carbon is reallocated from protein synthesis to polysaccharide storage. These techniques can enhance fermentable sugar yields for ethanol production processes [39].

Efficiently converting algal-derived carbohydrates into fermentable sugars requires adequate pretreatment and hydrolysis methods. Currently, several methods provide adequate pretreatment and hydrolysis for algal-derived carbohydrates. Enzymatic hydrolysis, dilute acid pretreatment, organogold pretreatment processes, and hybrid physicochemical pretreatment and hydrolysis are among the most common methods. Enzymatic saccharification is eco-friendly and provides effective hydrolysis due to its low temperature of about 35 °C, low pressure of less than 5 atm, and high-specificity sugar-only hydrolytic process. Using the integration of pretreatment with simultaneous saccharification/fermentation (SSF) reduces the inhibition associated with final product formation and improves the yields of ethanol produced [40,41].

New strategies for fermenting fuel ethanol, including high solids loading fermentations and the use of living yeast in held position fermentations, have been developed that increase capacity and lower operational costs. These types of fermentation improve how well microorganisms tolerate inhibitors, therefore producing higher concentrations of ethanol, making large scale production economically feasible [42,43,44].

Artificial intelligence-based optimization techniques such as response surface methodology (RSM) and neural networks have also recently been used to maximize carbohydrate accumulation during fermentation and optimize fermentation performance. By utilizing these data-driven methodologies, the ability to control the parameters associated with algal cultivation such as light intensity, pH, and nutrient concentrations have been substantially improved compared to conventional methods, thus increasing bioethanol production [45,46].

Integration of micro-algal cultivation with wastewater treatment systems provides an additional advantage by coupling bioethanol production with environmental remediation. Wastewater-derived nutrients support algal growth while simultaneously reducing organic load and nutrient pollution. The resulting biomass can be processed into fermentable sugars, supporting a multi-product biorefinery approach that enhances overall process sustainability [47].

## 7. Challenges and Recent Advances in Algal Biohydrogen Production

Despite its potential as a clean energy carrier, algal biohydrogen production remains limited by low yields and short-term operational stability. The major bottleneck is the oxygen sensitivity of hydrogenase enzymes, particularly [FeFe]-hydrogenases, which are responsible for hydrogen evolution but are rapidly inhibited by oxygen generated during photosynthesis. In addition, competition for photosynthetic electrons between biomass formation, carbon fixation, and hydrogen production restricts overall conversion efficiency. Recent strategies have focused on overcoming these limitations through metabolic engineering and cultivation-based approaches. Sulfur deprivation is one of the most widely investigated strategies, as it suppresses photosystem II activity and reduces oxygen evolution, creating a more favorable anaerobic environment for hydrogenase activity and sustained hydrogen production. However, sulfur limitation also reduces cellular growth and requires careful optimization to balance biomass formation and hydrogen generation. Genetic and metabolic engineering approaches, including modification of hydrogenase-related pathways and redirection of electron flow toward hydrogen evolution, represent promising strategies for improving productivity. Nevertheless, challenges related to low volumetric hydrogen yields, process stability, and large-scale reactor operation remain key barriers for industrial implementation of algal biohydrogen systems [48].

## 8. Biogas Production via Anaerobic Digestion

The anaerobic digestion (AD) process can convert microalgal biomass into methane-rich biogases and could provide significant value to excess biomass generated in biofuel production systems through its different uses production of methane-rich biogas. Different algal biofuel pathways exhibit distinct advantages and limitations depending on the biomass fraction utilized, conversion strategy, technological maturity, and economic feasibility. A comparative assessment of biodiesel, bioethanol, biogas, and biohydrogen production routes is provided in Table 2. After producing biodiesel or bioethanol via lipid or carbohydrate extraction, approximately 50–70% of the original algal biomass remains. Although established technology, various aspects, such as the biophysical composition of an algal cell wall, low carbon to nitrogen (C/N) ratios, and ongoing ammonia inhibition during digestion, limit the efficiency of AD as a viable pathway to producing methane. All of these factors can have detrimental effects on microbial activity and consequently affect the amount of methane produced [49,50].

There are multiple pretreatment strategies available to improve the biodegradability of microalgal biomass before performing AD. Numerous studies have evaluated various pretreatments, including enzymatic, thermal, alkaline, and mechanical, to disrupt the rigid cell wall structure of algae and enhance the solubilization of organic matter. Among the various methods, enzymatic hydrolysis has been particularly effective at enhancing the methane production of methanogenic microorganisms because they enhance microbial access to the substrate. However, excessive degradation of proteins can result in the accumulation of ammonia, which would inhibit methanogenesis when uncontrolled [51,52].

Recent advances in nanotechnology have introduced the use of nanomaterials as stimulants to enhance AD performance. Certain conductive nanomaterials, particularly iron-based nanoparticles, may enhance AD through improved electron transfer pathways, including direct interspecies electron transfer (DIET). However, other metal oxide nanoparticles, such as NiO and ZnO, may influence digestion through different mechanisms, including trace metal supplementation or enzyme regulation, while excessive concentrations may cause toxicity due to metal ion release [53,54].

The utilization of microalgal co-digestion with organic waste materials, including cattle manure, sewage sludge, and agricultural residues, is a viable means to address challenges associated with C/N ratios and increase biogas production through AD. This approach improves methane productivity by providing greater microbial diversity, buffering capacity stable operation of the AD system, and greater biogas production than mono-digestion systems [55,56].

Microalgae AD techno-governmental analyses indicate that the costs associated with producing energy through AD systems utilizing microalgal biomass will continue to remain elevated relative to traditional fossil-fuel-based energy systems. Nonetheless, improvements in biomass productivity, efficiency of pretreatment, and optimization of AD reactors are expected to significantly improve the economic viability of microalgal AD systems. Optimizing microalgal AD systems within the context of a microalgae biorefinery concept also provides additional sustainability to the entire system by making it possible to recover energy from the residual bio-mass streams produced from microalgae AD. One of the principal barriers to the practical application of microalgae AD on a commercial scale is process stability. Methane recovery is strongly influenced by operational parameters, including organic loading rate, ammonia concentration, and microbial community structure. Recent studies demonstrate that alterations in methanogenic pathways, i.e., hydrogenotrophic versus acetolactic, also play an important role in the recovery of AD reactors following disturbances and provide useful information for the chemical engineering of future commercial scale microalgal AD systems [57].

Pretreatment of algal biomass is a critical step for improving anaerobic digestion efficiency because the rigid cell walls of many algal species limit microbial accessibility to intracellular organic compounds. Mechanical pretreatments, including milling, ultrasonication, and high-pressure homogenization, enhance cell disruption and increase substrate availability; however, their application at large scale may be limited by high energy requirements. Thermal pretreatment, such as heat treatment and hydrothermal processes, improves biomass solubilization by disrupting cellular structures and accelerating hydrolysis, but excessive heat input may reduce the overall energy balance of the process. Chemical approaches, including alkaline and acidic treatments, can enhance the degradation of cell walls and increase methane recovery, although chemical consumption and potential inhibitor formation remain important considerations. Enzymatic pretreatment provides a more selective and environmentally compatible approach by targeting specific cell wall components, but the high cost of enzymes can restrict industrial implementation. Therefore, the selection of pretreatment strategy requires balancing improvements in methane yield with additional energy and operational costs, and integrated approaches combining pretreatment with algal biorefineries may provide a more economically feasible pathway [58].

**Table 2:** Comparative assessment of major algal biofuel pathways, highlighting differences in biomass components utilized, production yields, conversion efficiency, technology readiness level, economic feasibility, and key challenges associated with biodiesel, bioethanol, biogas, and biohydrogen production.

Biofuel Pathway	Main Biomass Component Utilized	Typical Yield/Performance Indicator	Conversion Efficiency	Technology Readiness Level (TRL)	Economic Considerations	Key Limitations	Reference
Biodiesel	Lipids	Lipid content typically 20–50% of dry biomass depending on species and cultivation conditions	High conversion efficiency through transesterification; influenced by lipid extraction efficiency	TRL 5–7 (pilot to demonstration stage)	High production cost due to cultivation, harvesting, and lipid extraction; improved by integrated biorefineries	Energy-intensive harvesting and downstream processing	[59]
Bioethanol	Carbohydrates	Carbohydrate content commonly 20–60% of dry biomass depending on strain and stress conditions	Dependent on hydrolysis and fermentation efficiency	TRL 4–6 (laboratory to pilot scale)	Lower feedstock competition but limited by pretreatment and fermentation costs	Efficient carbohydrate recovery and inhibitor formation	[60]
Biogas	Whole biomass (proteins, carbohydrates, lipids)	Methane production depends on biomass composition and pretreatment; reported energy recovery through anaerobic digestion	Mature anaerobic digestion technology with established conversion pathways	TRL 7–9 (commercially established for biomass digestion)	More economically feasible due to utilization of residual biomass after extraction	Low digestibility of algal cell walls and ammonia inhibition	[61]
Biohydrogen	Carbohydrates and organic compounds	Lower volumetric productivity compared with other pathways; dependent on light conversion and hydrogenase activity	Limited by oxygen sensitivity and low solar-to-hydrogen efficiency	TRL 3–5 (early development/pilot stage)	High future potential but currently limited by reactor design and production costs	Low productivity and scale-up challenges	[62]

## 9. Algae in Microbial Fuel Cells

### 9.1. Mechanism of Algae MFC Systems

Algal biocathodes generate oxygen through photosynthesis and can serve as the terminal electron acceptor at the cathode while simultaneously contributing to carbon fixation and biomass production. Photosynthetic microbial fuel cells represent a broader category in which light-driven biological processes are integrated with bioelectrochemical energy recovery, and algae may participate either as cathodic oxygen producers or as components of the overall photosynthetic system. Therefore, differentiating these configurations is essential for accurately evaluating the mechanisms, advantages, and limitations of algae-integrated MFC technologies [63].

Photosynthetic microbial fuel cells (PMFCs) exhibit unique electrochemical behavior due to the light-dependent activity of algal biocathodes. During illumination, photosynthetic oxygen evolution increases dissolved oxygen availability at the cathode, enhancing the oxygen reduction reaction (ORR) and improving cathodic electron acceptance. Conversely, dark periods reduce oxygen production and may decrease cathode potential and overall voltage output. Therefore, algal circadian rhythms and operational light/dark cycles represent critical factors controlling PMFC performance. The electrochemical efficiency of PMFCs is also influenced by cathode configuration, algal species, electrode material, and reactor design. Although platinum-based abiotic cathodes generally provide high catalytic activity, advanced photosynthetic cathode configurations have demonstrated competitive power outputs by combining oxygen generation, carbon fixation, and bioelectrochemical energy recovery. Further optimization of light management, electrode architecture, and algal metabolic activity is required to achieve stable and scalable PMFC performance [64].

### 9.2. Algae at the Cathode: Biocathode MFCs

A significant amount of research is being done on algae being used for photosynthetic biocatalysis on the cathode of MFCs because algae’s ability to produce dissolved oxygen in the cell gives energy to the cathodic oxygen reduction reaction and lets the algae sequester CO_2_ at the same time. Algal biocathodes were reported in several recent articles as being an excellent way to link bioelectricity generation and nutrient reclamation, as well as to treat wastewater, to support a circular bioeconomy [65]. The incorporation of microalgae into biocathodes has improved oxygen availability as well as electrical generation capacity. For example, an improvement was reported in oxygen reduction kinetics and cathodic performance of a three-chamber plant microbial fuel cell with *Chlorella sorokiniana*, resulting in stable self-sustained electric power generation [9].

In a comprehensive evaluation of the cathodic microorganisms in photosynthetic MFCs, it was determined that the selection of the algal species for the cathode has a substantial effect upon electrochemical performance, oxygen production, and wastewater treatment efficiency. This study suggested that optimizing the cathodic microbial communities is critical to establishing the long-term stability and scalability of PMFCs [66]. The results of the recent use of *Chlorella vulgaris* in photosynthetic microalgae MFCs, where improved electric power generation, ammonium assimilation, and enhanced lipid productivities were all reported, demonstrate the dual benefits of generating energy and increasing the value of biomass [67].

A recent review specifically revisiting algal biocathodes concluded that these systems not only improve bioelectrogenesis but also facilitate nutrient recovery, carbon sequestration, and heavy metal remediation, highlighting their growing importance in sustainable wastewater treatment technologies [68].

### 9.3. Algae at the Anode: Direct Electron Donors

Microalgae can act as electron donors in anode of MFCs directly or indirectly through the biodegradation of their harvested biomass. There is also evidence that microalgae-derived biomass is a good energy-rich substrate to support electroactive microbial populations, to aid recovery of resources, and produce carbon-neutral energy. Including microalgal biomass in MFC systems has been shown to not only improve anodic bioelectrochemical activity of electroactive populations but also improve biodegradability of the substrates and increase electron recovery efficiencies resulting in better overall MFC performance [69]. Further studies have examined the use of pretreated microalgal biomass as anodic substrates to generate increased current output, higher coulombic efficiencies, and better organic removal than conventional substrates. Results from these studies demonstrate significant variability in electron transfer efficiencies, and overall power outputs are due to the composition of the biomass and the pretreatment methods employed [70].

Interest in algae-based anodic systems is increasing, likely due to the high levels of lipids and carbohydrates available in microalgal biomass, their rapid reproductive cycle, and the fact that microalgal biomass can be cultivated in a wastewater system. The integration of biomass production, wastewater treatment, and bioelectricity generation into a unified system has been proposed to improve both environmental sustainability and the economic viability of bioelectricity generation using microalgal biomass [71]. More recently, cascading bio-electrochemical systems have been developed that process harvested microalgal biomass to extract valuable compounds for subsequent use as anodic substrates. The integration of these types of systems increases the overall efficiency of energy recovery, reduces dependence on outside carbon sources, and supports the establishment of a circular bioeconomy. Future research efforts are expected to emphasize optimizing biomass pretreatment methods, engineering electroactive microbial communities, and improving reactor design to facilitate scale-up of these bio-electrochemical systems [72].

### 9.4. Integrated and Cascade MFC Designs

MFC technology has progressed to utilize more complex design structures as a way to maximize simultaneous bioelectricity, wastewater treatment, carbon sequestration and biomass production in the same system. Findings from several recent studies have shown that two to three stage cascade type photosynthetic MFC configurations (e.g., CA-MFCs) could greatly improve the performance of these systems by optimizing hydraulic retention time, nutrient availability and oxygen production. These two- to three-stage CA-MFC configurations have produced higher power output, a greater amount of lipidaceous biomass accumulation, and greater efficiencies in removing organic matter, nitrogen, and phosphorus. Therefore, they represent an excellent potential for combined wastewater treatment and resource recovery [73].

Another key strategy for enhancing the performance of photosynthetic MFCs is the optimization of interactions among microbial communities. Synergistic interactions between electroactive bacterial consortia and photosynthetic microalgae have been shown to result in improved efficiency of electron transfer, higher levels of cathodic oxygen availability, and improved stability of the overall system. Recent efforts suggest that engineering the microbial consortia may represent a more sustainable alternative to enhancing power density than merely modifying the electrode [69].

Recently, the potential for integrated microbes and pond algae growing on carbon-rich wastewater has come to the attention of researchers and industry using bio-electrochemical systems. These ecosystems enable concurrent carbon saturation, electricity generation, and removal of pollutants without the need to add carbon from outside the system. Earlier studies indicate that integrated algae and bacteria electroactive systems improve remediation of heavy metals, especially Cr-contaminated wastewaters, all while providing stable bioelectricity production, maintaining sufficient power to operate the entire system when exposed to sunlight. Adding high-valued commercially grown microalgal species to PMFCs allows for further application of these systems. In addition to creating electricity, the photosynthetic cathodes associated with PMFCs can support the synthesis of metabolites intermediate product used to make some kind of more finished product for commercial sale, such as carotenoids, chlorophyll, antioxidants, and fats or oils. The integrated biorefinery concept widens the scope for economic viability of PMFC technologies through coupling cleanup of the environment with the creation of sellable bioproducts [66,72].

Despite significant advances in algae-integrated microbial fuel cells, their transition from laboratory-scale demonstrations to industrial applications remains challenging. Most reported algae MFC systems are evaluated under controlled conditions using small reactors, optimized substrates, and short operational periods, which may not accurately represent large-scale performance. One major limitation is the high cost of electrode materials, particularly when advanced catalysts or conductive materials are required to achieve enhanced electrochemical activity. Developing low-cost, durable, and scalable electrode materials is therefore essential for commercial implementation. Membrane-related challenges also represent a critical barrier, as membrane fouling caused by biomass accumulation and extracellular polymeric substances can increase internal resistance and reduce long-term performance. Moreover, maintaining stable operation over extended periods remains difficult due to changes in algal activity, microbial community structure, and electrode performance. Large-scale systems must also overcome engineering challenges associated with light distribution, mass transfer, oxygen management, and reactor design. Future progress will require integrated approaches combining low-cost materials, optimized reactor architectures, membrane-free configurations, and long-term operational validation to achieve economically feasible algae MFC platforms [74].

**Figure 4:**
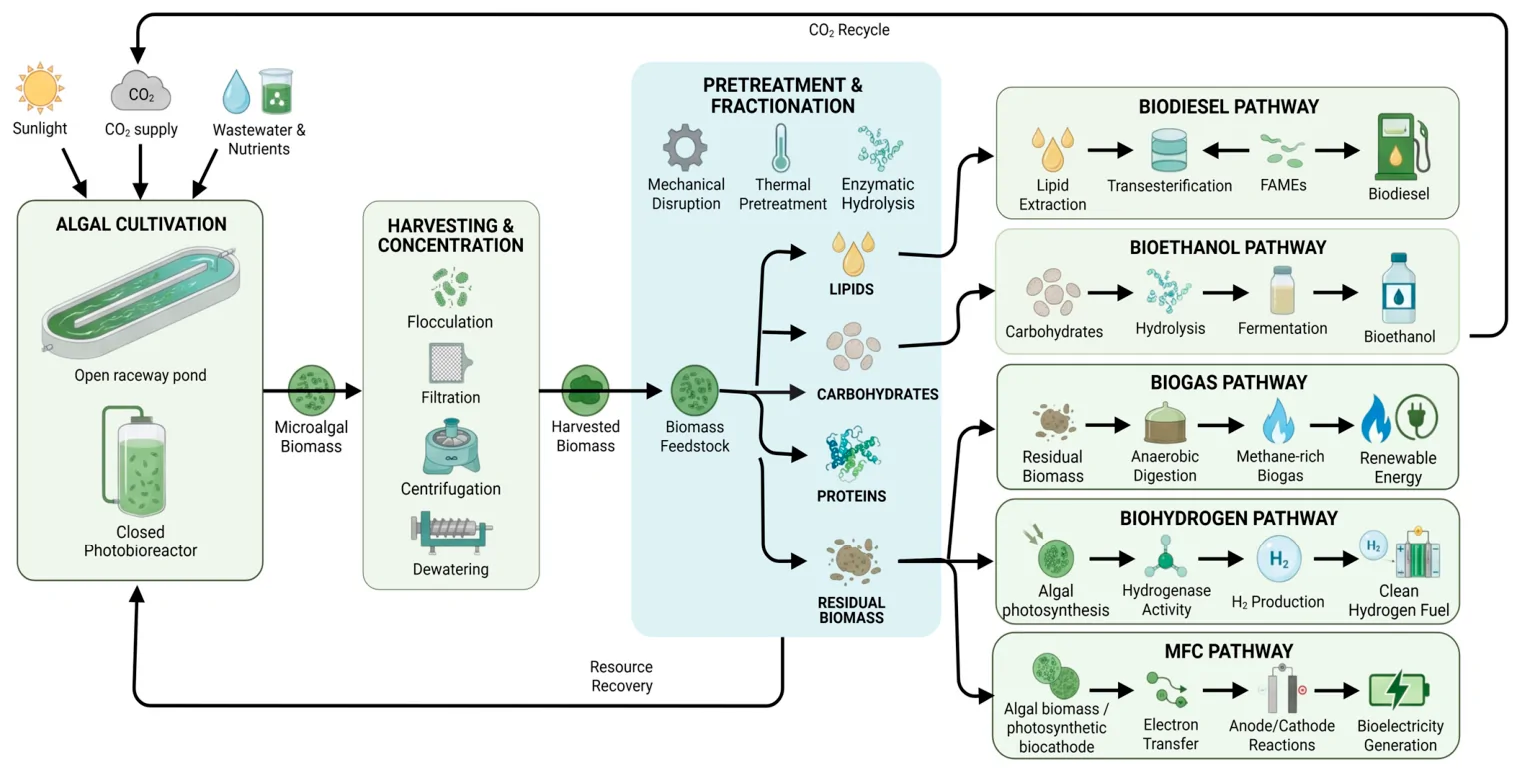
Integrated process flow diagram of algae-based bioenergy systems. The schematic illustrates the complete algal biorefinery workflow, including algal cultivation, biomass harvesting and concentration, pretreatment and fractionation of biomass components, and their conversion into multiple renewable energy products. Lipid, carbohydrate, and residual biomass fractions are directed toward biodiesel, bioethanol, biogas, biohydrogen, and algae-integrated MFC pathways, while CO_2_ recycling and resource recovery highlight the circular bioeconomy potential of integrated algal systems.

Electron transfer mechanisms represent a critical determinant of the performance of algae-integrated MFCs. In these systems, electrons generated from algal metabolism can reach the electrode through direct or mediated pathways. Direct electron transfer occurs when algal cells or associated electroactive microorganisms establish physical contact with the electrode surface, allowing electrons generated during metabolic reactions to be transferred through membrane-associated components or conductive extracellular structures. Alternatively, mediated electron transfer involves soluble electron carriers that shuttle electrons from biological cells to the electrode. These mediators may originate from cellular metabolites or be externally supplied compounds that facilitate electron transport and enhance current generation. In algae-based MFCs, electron transfer is further influenced by the role of algae as photosynthetic biocathodes, where oxygen generated during photosynthesis supports cathodic reactions, or as biomass substrates where algal organic compounds are oxidized by electroactive microorganisms at the anode. Understanding and optimizing these electron transfer pathways is essential for improving power output and scalability of integrated algal MFC systems [75]. The integration of lipid, carbohydrate, and residual biomass conversion pathways enables the simultaneous production of biodiesel, bioethanol, biogas, biohydrogen, and bioelectricity through microbial fuel cells, as illustrated in Figure 4.

Recent research has also investigated the use of unique reactor designs that do not use membrane exchange processes or use buffer solutions. The simplicity of these designs lowers the operating cost of PMFCs, as they do not require costly membrane systems and chemical buffers to produce sufficient bioelectricity and to have efficient treatment of the wastewater. These developments have placed the commercialization of photosynthetic microbial fuel cells on the pathway to practical, large-scale application [76]. The performance characteristics of algae-assisted microbial fuel cell systems and their applications in bioelectricity production and wastewater treatment are summarized in Table 3.

**Table 3:** Comparative performance of algae-integrated microbial fuel cell systems, highlighting algal species, MFC configurations, functional roles, key performance indicators, and technological outcomes in bioelectricity generation, resource recovery, and wastewater treatment.

Algal Species/Consortium	MFC Configuration	Main Function of Algae	Performance Indicators	Key Outcomes	Reference
*Chlorella sorokiniana*	Three-chamber photosynthetic microbial fuel cell with algal biocathode	Oxygen production for cathodic oxygen reduction and simultaneous biomass generation	Power generation, cathodic performance, system stability	Improved oxygen availability and enhanced electrochemical performance through photosynthetic biocathode integration	[8]
*Chlorella vulgaris*	PMFC	Algal biocathode for oxygen production, nutrient assimilation, and biomass recovery	Power output, ammonium removal, lipid productivity	Demonstrated combined energy generation and biomass valorization, supporting multifunctional algal MFC platforms	[47]
Microalgal consortia	Algal–bacterial photosynthetic MFC	Synergistic interaction between algae and electroactive bacteria	Bioelectricity generation, nutrient removal, carbon fixation	Improved system sustainability through enhanced microbial interactions and simultaneous wastewater treatment	[46]
Microalgal biomass (various species)	Anodic MFC configuration using algal-derived substrates	Electron donor through degradation of lipid- and carbohydrate-rich biomass	Current density, coulombic efficiency, organic matter removal	Pretreated algal biomass improved substrate availability and electron recovery efficiency compared with conventional substrates	[49,50]
*Chlorella* spp.	Integrated algae–MFC wastewater treatment system	Coupling wastewater remediation with bioelectricity production	COD removal, nutrient recovery, power production	Demonstrated the potential of integrating biomass production, pollutant removal, and energy recovery within a circular bioenergy framework	[51]
Algal–bacterial consortium	Cascade photosynthetic MFC system	Integrated oxygen production, nutrient cycling, and biomass accumulation	Power output, lipid accumulation, nitrogen and phosphorus removal	Multi-stage configurations improved overall energy recovery and wastewater treatment efficiency	[53]
Photosynthetic algal communities	Membrane-free photosynthetic MFC designs	Simplified bioelectrochemical operation using algal oxygen production	Operational stability, energy recovery, treatment efficiency	Reduced operational complexity and improved potential for large-scale implementation of algal MFC systems	[76]

### 9.5. Algae–Bacteria Synergistic Systems

Algae–bacteria consortia represent an important strategy for improving the performance of integrated algal bioenergy systems by combining the complementary metabolic activities of both microorganisms. During photosynthesis, algae provide oxygen and fixed carbon compounds that support bacterial metabolism, while bacteria release CO_2_ through respiration and contribute to organic matter degradation and nutrient recycling, creating a mutually beneficial interaction. These synergistic relationships can enhance biomass productivity, wastewater nutrient removal, and bioenergy recovery. However, maintaining stable algae–bacteria communities remains challenging due to competition for nutrients, changes in environmental conditions, bacterial overgrowth, and imbalance in oxygen availability. Long-term stability requires careful management of operational parameters, including light intensity, nutrient composition, pH, microbial inoculation ratios, and reactor configuration. Strategies such as controlled co-cultivation, immobilized systems, and biofilm-based reactors may improve community stability and facilitate the scale-up of algae–bacteria integrated bioenergy platforms [77].

## 10. Integrated Algal Biorefinery and Wastewater Treatment

The biorefinery concept valorizing multiple products from a single algal biomass feedstock has become a central strategy for improving the economic feasibility of algal bioenergy systems. Modern integrated algae biorefineries are designed to couple biofuel production with the recovery of high-value compounds such as pigments, proteins, and nutraceuticals. Recent literature highlights that economic viability depends strongly on integrating upstream cultivation with downstream fractionation and conversion processes, including thermochemical and biochemical pathways, supported by advances in strain engineering and process optimization [78].

Wastewater-based algal cultivation has emerged as a key enabling platform for sustainable biorefineries. Algae-based systems efficiently remove nutrients such as nitrogen and phosphorus while simultaneously generating biomass suitable for energy and bioproduct applications. The integration of algae with bacterial consortia further enhances treatment efficiency, system stability, and CO_2_ fixation, making such hybrid systems increasingly important for circular wastewater-to-energy frameworks [79].

Hydrothermal liquefaction is recognized as one of the most promising thermochemical conversion routes for wet algal biomass, eliminating the need for energy-intensive drying. This process enables direct conversion of biomass into biocrude with high energy density and favorable carbon recovery. Recent studies report improved energy efficiency and scalable potential when HTL is applied to wastewater-grown microalgae systems, positioning it as a leading candidate for next-generation algal biofuel production platforms [80].

## 11. Nanotechnology-Enhanced Algal Bioenergy Systems

### 11.1. Nanomaterials for Algal Cultivation Enhancement

Nanotechnology is being utilized as an avenue to support algal bioenergy by providing solutions for some of the major weak points in the algal biofuel chain, such as slow biomass accumulation, high harvesting costs, and inefficient lipid conversion, as well as limited reactor productivity. In algal systems, nanomaterials have numerous potential applications such as stimulating algal growth, improving light management strategies, enhancing the delivery of nutrients, flocculating algal biomass, providing catalytic surfaces, and modifying electrode performance. However, the use of nanotechnology does not guarantee benefits. For example, a nanoparticle that may provide productivity benefits at a lower concentration may inhibit photosynthesis, damage membrane structures, or cause downstream contamination issues at higher concentrations. As such, nanotechnology serves as an optimizer when working with algal bioenergy, rather than providing a solution [81].

Specifically, the use of nanoparticles, including iron oxide, zinc oxide, titanium dioxide, silica, carbon nanotubes, graphene derivatives, and magnetic nanoparticles, has been studied to enhance algal growth and improve algal nutrient uptake, CO_2_ utilization, and lipid accumulation. Magnetic nanoparticles are particularly compelling since they can support both algal biomass cultivation and harvesting through magnetic separation. Some nanoparticles have demonstrated the ability to increase light scattering within photobioreactors, thus improving algal photosynthetic efficiency; however, caution should be employed when working with excessive levels of nanoparticles as they may lead to oxidative stress, reduce chlorophyll levels, and inhibit algal growth. In practical applications, the effects from nanoparticles are dependent upon several factors, including dose, particle size, surface coating, and the specific algal strain [82].

### 11.2. Nanomaterials-Assisted Harvesting and Biomass Recovery

The economic and environmental feasibility of nanoparticle-assisted algal bioenergy systems depends not only on nanoparticle performance but also on efficient recovery and reuse strategies. Magnetic nanoparticles, such as iron oxide-based materials, offer advantages due to their facile separation using external magnetic fields, enabling repeated utilization and reducing material loss. Other nanomaterials may require alternative recovery approaches, including centrifugation, filtration, or immobilization onto supporting matrices such as carbon-based materials or polymeric carriers. Immobilized nanomaterials can minimize nanoparticle release into aquatic environments while improving operational stability. However, repeated reuse may be limited by nanoparticle aggregation, surface fouling by algal biomass, and gradual loss of catalytic or functional activity. Therefore, future development of nanoparticle-enhanced algal systems should prioritize recoverable, recyclable, and environmentally compatible nanomaterials to improve economic feasibility and reduce ecological risks [83].

Nanotechnology-assisted harvesting strategies can overcome the limitations of conventional microalgal biomass separation by exploiting surface interactions between engineered nanoparticles and algal cells. Microalgal cells generally exhibit negatively charged surfaces due to functional groups present in their extracellular matrix, resulting in negative zeta potentials that hinder spontaneous aggregation. Surface modification of magnetic Fe_3_O_4_ nanoparticles with positively charged polymers, such as chitosan or polyethyleneimine, enhances electrostatic attraction between nanoparticles and algal cells, promoting efficient flocculation and subsequent magnetic recovery. Furthermore, advanced surface functionalization approaches, including silane-based modification, can improve nanoparticle stability, facilitate separation at the water–solvent interface, and enable repeated recovery and reuse of magnetic nanomaterials. These strategies provide a pathway toward reducing harvesting costs and improving the sustainability of nanoparticle-assisted algal biomass recovery systems [84].

### 11.3. Nanomaterials for Catalytic Conversion of Algal Biomass

When converting algal lipids to biodiesel, nanocatalysts can also provide essential support. Conventional transesterification often relies on homogeneous catalysts consisting of either an acid or base and, when using these methods, many problems can arise such as separation, corrosion and/or soap formation; and during transesterification, wastewater will be a byproduct. Heterogeneous nano catalysts, such as metal oxides, magnets, calcium oxide nanoparticles, and carbon-based nanoparticles, provide a greater surface area than homogeneous catalysts, allow for greater reusability, and provide more ease of recovery. The importance of a heterogeneous nano catalyst will directly relate to the presence of water, pigment, and free fatty acids found in algal oil that can interfere with the conversion. Even though nanocatalysts can enhance the reaction efficiency, their function depends directly on the stability, regeneration, cost, and rate of deactivation of the catalyst throughout multiple production cycles [85].

### 11.4. Nanomaterial-Modified Electrodes for Algae-Integrated MFC Systems

Nanomaterials have also emerged as promising tools for improving the electrochemical performance of algae-integrated MFC systems through electrode modification. Carbon-based nanomaterials, including graphene, graphene oxide, and carbon nanotubes, as well as metal oxide nanomaterials, can enhance electrode properties by increasing surface area, improving electrical conductivity, and facilitating electron transfer between biological components and electrode surfaces. These modifications can promote microbial attachment, improve biofilm formation, and reduce charge transfer resistance, thereby enhancing current generation and overall power output. In algae-based MFC configurations, nanostructured electrodes may further support photosynthetic biocathode performance by improving oxygen reduction reactions and maintaining efficient electron transport pathways. However, large-scale implementation remains limited by several challenges, including the high cost of advanced nanomaterials, complex fabrication procedures, potential nanoparticle release, electrode fouling, and long-term stability under continuous operation. Therefore, future research should focus on developing low-cost, durable, and environmentally compatible nanostructured electrodes that maintain enhanced electrochemical activity while enabling scalable integration into algae-based bioenergy systems [86].

Nanostructured carbon electrodes can significantly improve the electrochemical performance of algae-integrated MFC systems by modifying electrode architecture and catalytic properties. Carbon-based supports, including graphene, carbon nanotubes, and porous carbon materials, provide high surface areas and interconnected conductive networks that enhance biofilm attachment, electron transport, and charge transfer efficiency. Furthermore, the dispersion of catalytic nanoparticles onto carbon supports can increase the availability of active sites while preventing nanoparticle aggregation, thereby improving electrode stability and catalytic activity. Metal–carbon interactions may further regulate electronic properties at the electrode interface, facilitating more efficient electrochemical reactions. These principles, widely investigated in carbon-supported electrocatalysts for fuel-cell applications, provide valuable insights for designing advanced nanostructured electrodes for bioelectrochemical energy systems [87]. A detailed comparison of the relevant features, performance indicators, and technological aspects is provided in Table 4.

Nanomaterials have also found application in MFCs; this includes algal and photosynthetic MFCs. These systems can use the oxygen generated via photosynthesis from algae as well as providing a means for algae to assist in treating wastewater. Additionally, algae will also contribute to the generation of bioelectricity. Nanostructured MFC electrodes using materials such as graphene, carbon nanotubes, metal oxides, or conductive polymer composites will help to increase the surface area of the electrodes, thus enhancing the ability of the microbes to attach, improving electron transfer and ultimately increasing the power density. Even though the advances in nanomaterials to improve MFC performance show great promise, the overall performance is still limited due to low current output, difficulty with scaling and electrode fouling, and the high cost of the reactors. Therefore, modified MFCs using nanomaterials are more likely to be viewed as emerging hybrid wastewater–energy platforms as opposed to mature electricity generation technologies [94].

The synthesis of nanoparticles with algae uses algal biomass and algal extracts as reducing agents in conjunction with biocompatible nanomaterials. Using algae to produce nanomaterials, this green synthesis method will allow for the reduction of harmful chemical agents necessary to produce nanoparticles and enable the creation of biocompatible nanoparticles. Nanoparticles can have significant catalytic, antifungal, or environmental value and are derived from both micro- and macroalgae. In the case of biofuels produced from algae, this offers a closed loop where algae produce biomass while providing the nanomaterials needed to support the cultivation and harvesting of algae, catalysis to convert the biomass to biofuel, and anaerobic digestion for wastewater treatment [95].

The integrated nanotechnology-enhanced bioenergy systems in the algae production process show great promise for the interconnection between algae cultivation, harvesting, converting to biofuel, and bio-electrochemical conversion to produce energy and energy storage as a comprehensive approach. Unfortunately, most of the studies conducted on algae and nanoparticles occur at the laboratory scale, and it is still uncertain how to translate these results to an industrial scale. Therefore, the major areas of concern still unresolved are (1) the cost of nanoparticles; (2) the potential for environmental release of nanoparticles; (3) the potential for toxicity of nanoparticles to algae; (4) if catalysts containing nanoparticles can be recovered; (5) if the life cycle of biofuels and nano-biofuels can be sustained; and (6) if there will be acceptance from regulating agencies for nanoparticles in environmental applications. Developing resilient designs supports the use of recyclable, biodegradable, magnetic, or intrinsically derived algae as the source of nanomaterials rather than just artificially engineered nanoparticles. The ultimate direction of the nanotechnology-algae biofuels program will depend on demonstrating the added benefit of nanoparticles across multiple biofuel-related functions, rather than solely for one functionality [96]. Recent studies (2023–2026) demonstrate the growing role of nanotechnology in improving algal bioenergy systems, including enhanced biomass production, increased lipid accumulation, improved catalytic efficiency in biodiesel production, and optimized nanomaterial-assisted bio-electrochemical systems, as summarized in Table 5.

### 11.5. Mechanisms of Nano-Enabled Enhancement in Algal Systems

The effects of nanomaterials on algal performance are governed by their physicochemical properties, including composition, size, surface charge, catalytic activity, and interaction with algal cells. Iron oxide nanoparticles can enhance algal growth by providing bioavailable iron, an essential element involved in photosynthetic electron transport and enzymatic processes, while their magnetic properties also facilitate biomass recovery. Zinc oxide nanoparticles may improve cellular metabolism by supplying zinc ions required for enzymatic functions; however, excessive concentrations can induce reactive oxygen species (ROS) generation and inhibit photosynthesis. Titanium dioxide nanoparticles can influence light utilization through photocatalytic and light-scattering properties, potentially improving photosynthetic efficiency under controlled conditions. Carbon-based nanomaterials, including carbon nanotu and graphene derivatives, enhance electron transfer, surface interactions, and conductivity, which can support photosynthetic activity and bioelectrochemical applications. Therefore, the beneficial or inhibitory effects of nanomaterials depend strongly on nanoparticles’ physicochemical characteristics, exposure concentration, and algal species-specific responses [101].

## 12. Challenges and Future Perspectives

Even with the great potential that algae bioenergy, as well as MFCs, can present, there are still many technical and economic challenges that limit their expansive use and adoption. The main cost limiting the wide-scale use of algae to produce biofuels is the overall cost of everything involved in that process, from growing the algae to harvesting it and eventually extracting the oil. It has been recently shown that there are very few types of algae that exhibit the ideal characteristics for biofuel production in terms of a combination of high oil yield, rapid growth rate, and being resilient and adaptable to industrial production environments [102,103].

Harvesting and dewatering algae are two of the most energy-demanding operations when viewed as part of the entire process following the growing of the algae until the extraction of the oil. Additionally, the current inefficiency of the recovery of oil and separation of biomass will continue to increase the total cost to produce algae-based biofuels, which currently equates to algae-based biofuels being less cost-effective than fossil-fuel based alternatives. Various proposed methods to overcome these limitations include flocculation with low-energy methods, utilizing bio-based coagulants during harvesting, and utilizing integrated systems of grow–harvest/single species-coculture/mixotrophic and co-locating cultivation systems to improve total process validity and efficiency [104].

Multiple operational parameters that are linked together, including light intensity, pH stability, temperature, and nutrient metering availability, affect the performance of microbial fuel cell systems; therefore, there have been extensive efforts towards optimizing parameters; however, MFCs with algae in terms of power output are still significantly lower than that required for practical use, likely due to the resistive losses within the MFC and poor electron transfer efficiency. Thus, it is apparent that there is a requirement for new materials to construct electrodes from as well as scalable designs for reactors. When focused on anaerobic digestion systems with microalgal substrates, one of the other major limitations is in the process stability of these systems. Previous modelling studies have shown that most conventional frameworks based on the ADM1 are incapable of operating under overload conditions. Due to these results, improved predictive modelling accuracy requires the use of inhibition assumptions associated with accumulation of volatile fatty acids and metabolic stress during transient periods [105].

At the cellular level are the genetic and metabolic engineering features which represent an opportunity for overcoming limitations experienced by algal bioenergy production systems. Advances in synthetic biology now allow targeted approaches to genetic modification of lipid biosynthesis, carbon fixation efficiency, and stress tolerance to increase yield achieved through providing algae with a better ability to capture solar energy for photosynthesis, which increases biomass yields. In addition to this, strain optimization strategies are also being evaluated for hydrogen production. The microalgae Scenedesmus spp. are also being assessed for their utility in phytoremediation activities and hydrogen production when combined with other waste sources [106].

Techno-economic analysis (TEA) indicates that the commercial feasibility of algae-based bioenergy systems remains primarily constrained by high cultivation, harvesting, and downstream processing costs. Cultivation represents a major fraction of overall expenses due to the requirements for reactor infrastructure, nutrient supply, CO_2_ delivery, and energy-intensive mixing. In addition, biomass harvesting and dewatering remain critical economic bottlenecks because of the low cell density of algal cultures and the high energy demand associated with conventional separation techniques. Downstream conversion processes, including lipid extraction, transesterification, and biomass pretreatment, further contribute to production costs. Recent TEA studies suggest that integrated algal biorefineries incorporating wastewater treatment, CO_2_ utilization, and recovery of valuable co-products can improve economic viability by distributing costs across multiple revenue streams [107].

For algae-integrated microbial fuel cell systems, economic challenges are mainly associated with electrode materials, membranes, reactor construction, and limited electricity generation rates. Although MFCs provide simultaneous wastewater treatment and energy recovery, their low power density and high material costs currently restrict large-scale commercialization. Future improvements in low-cost electrode materials, scalable reactor designs, and integration with other algal conversion pathways will be essential for achieving economically competitive algae-based bioenergy systems [108]. Life-cycle assessment (LCA) provides a comprehensive framework for evaluating the environmental sustainability of algae-based bioenergy systems by considering all stages from cultivation and biomass production to conversion and final product utilization. Although algae can contribute to greenhouse gas mitigation through biological CO_2_ fixation, the overall carbon balance depends strongly on energy inputs associated with cultivation, mixing, harvesting, drying, and downstream processing. Several LCA studies have demonstrated that the environmental benefits of algal biofuels are highly dependent on process optimization, renewable energy integration, and utilization of waste-derived resources [24].

Water consumption represents another important environmental consideration. Conventional algal cultivation may require significant water inputs; however, the use of wastewater and saline water resources can reduce freshwater demand while simultaneously providing nutrient removal services. In addition, algae-based systems generally offer advantages in land use efficiency compared with terrestrial biofuel crops because they can be cultivated on non-arable land and within controlled photobioreactor systems. Nevertheless, infrastructure requirements and energy consumption associated with large-scale cultivation must be considered when evaluating the overall environmental performance. Therefore, future development of algae-based bioenergy systems should focus on integrated biorefineries, resource recycling, renewable energy inputs, and optimized process design to maximize environmental benefits and achieve favorable life-cycle impacts [109].

Although algae-based bioenergy technologies have demonstrated promising results at laboratory scale, their transition toward pilot- and demonstration-scale applications remains limited by several technical and economic barriers. Among different platforms, algal cultivation systems using open ponds and photobioreactors have reached pilot-scale demonstrations; however, maintaining consistent biomass productivity under outdoor conditions remains challenging due to contamination, environmental fluctuations, and energy requirements for mixing and operation. Biodiesel production from algal lipids has also progressed toward pilot-scale evaluation, but large-scale implementation is still restricted by high biomass harvesting costs, energy-intensive lipid extraction, and unfavorable economic performance compared with conventional fuels. In contrast, anaerobic digestion of algal biomass represents one of the more mature conversion routes, although challenges related to biomass recalcitrance and pretreatment requirements remain. Biohydrogen production and algae-integrated microbial fuel cell systems are generally at earlier developmental stages, with most studies conducted at laboratory scale due to limitations associated with low productivity, reactor design, electrode costs, and long-term operational stability. Therefore, future commercialization will require validation under realistic operating conditions, development of scalable reactor designs, reduction of energy inputs, and integration of multiple processes within algal biorefinery frameworks [108].

Biohydrogen production from microalgae represents a promising third-generation biofuel pathway that integrates solar energy conversion with sustainable hydrogen generation. Unlike conventional thermochemical hydrogen production routes, algal biohydrogen relies on biological processes in which photosynthetic microorganisms utilize light energy to drive hydrogen evolution while simultaneously contributing to carbon fixation. The major biological pathways for algal hydrogen production include direct biophotolysis, indirect biophotolysis, and photofermentative processes, which are primarily controlled by the activity of hydrogenase and nitrogenase enzymes. In direct biophotolysis, photosynthetic electrons generated during water splitting are transferred to hydrogenase enzymes, resulting in hydrogen production; however, this pathway is strongly limited by the sensitivity of hydrogenase enzymes to the oxygen generated during photosynthesis [110].

One of the most investigated strategies to enhance algal hydrogen production is sulfur deprivation, which temporarily restricts photosynthetic oxygen evolution and redirects cellular metabolism toward anaerobic conditions favorable for hydrogenase activity. Under sulfur-limited conditions, reduced photosystem II activity decreases oxygen accumulation, allowing hydrogenase enzymes to remain active for longer periods and improve hydrogen generation. In addition, metabolic engineering approaches, including modification of hydrogenase expression, regulation of electron transport pathways, and optimization of carbon partitioning, have been explored to improve hydrogen productivity and overcome inherent biological limitations [111].

Despite its potential, large-scale implementation of algal biohydrogen remains challenging due to several limitations, including low hydrogen productivity, oxygen sensitivity of hydrogen-evolving enzymes, complex reactor requirements, and difficulty maintaining stable production under outor conditions. Moreover, efficient light penetration, nutrient management, and separation between biomass growth and hydrogen production phases are critical factors affecting process performance. Recent advances in synthetic biology, genetic engineering, two-stage cultivation strategies, and optimized photobioreactor designs provide promising opportunities to improve hydrogen yields and operational stability. Future integration of biohydrogen production within algal biorefineries, where residual biomass can be valorized through complementary pathways such as anaerobic digestion or bioelectrochemical systems, may enhance the overall economic and environmental feasibility of algae-based hydrogen technologies [112].

## 13. Conclusions

This review article discusses the advancements achieved in algal-based bioenergy platforms from utilizing biomass resources for a single process to integrating these systems in bioenergy generation, resource recovery, and environmental management. Algae are considered versatile biological resources that can convert solar energy and carbon dioxide into biomass. Algae play an important role in the circular bioeconomy through wastewater treatment and creating many other biological products. Recent developments in algal microbial fuel cells expanded their function beyond supplying oxygen by creating multi-functional bioelectrochemical systems that generate bioelectricity, recover nutrients, and bind carbon, as well as convert biomass. Despite the considerable progress made in this area, the pathway for achieving industrial-scale applications of integrated algal bioenergy systems from laboratory results has not been smooth, owing to issues of economic viability, scalability, sustainability, and process efficiency. In this context, any claims made about improvements in material conversion or power production should be treated cautiously, considering that the resultant processes cannot be expected to perform equally well in reality to how they were developed in a controlled environment. Nonetheless, integrated algal biorefineries appear to be very promising since they can help attain improved functionality of the proposed systems through the maximization of all forms of biomass, lipids, carbohydrates, proteins, pigments, and residual biomass, while minimizing waste. It is also important to point out that successful commercialization can only be achieved if the innovative nanostructures, designed microbial consortia, scalable industrial reactors, and techno-economic aspects are properly combined.

## Figures and Tables

**Table 1:** Comparative CO_2_ fixation performance of representative algal species under different cultivation conditions, highlighting the influence of algal strain, cultivation system, and carbon availability on carbon sequestration efficiency.

Algal Species	Cultivation System/Conditions	CO_2_ Source or Cultivation Condition	CO_2_ Fixation Rate (g CO_2_/g Biomass/Day)	Key Characteristics	Reference
***Chlorella vulgaris***	Closed photobioreactor	CO_2_-enriched cultivation conditions	1.83	High growth rate and efficient carbon assimilation; widely investigated for bioenergy and wastewater applications	[26]
***Scenedesmus obliquus***	Photobioreactor cultivation	Elevated CO_2_ concentration	1.60	High biomass productivity and tolerance to increased CO_2_ levels	[27]
***Nannochloropsis oculata***	Controlled photobioreactor	CO_2_ supplementation with optimized nutrients	0.90	Marine microalga with high lipid productivity and carbon capture potential	[28]
***Chlorella pyrenoidosa***	Open cultivation system	Atmospheric CO_2_ conditions	0.75	Suitable for large-scale cultivation due to robust growth and environmental adaptability	[29]
***Spirulina platensis***	Open raceway pond	CO_2_-enriched air supply	0.65	High biomass productivity and suitability for outdoor cultivation systems	[30]
**Mixed microalgal consortium**	Wastewater-based cultivation	Industrial wastewater nutrients and CO_2_ availability	0.40–1.20	Combines carbon fixation with nutrient removal and wastewater remediation	[25]

**Table 4:** Reported effects of different nanoparticle types on representative algal species, highlighting tested concentration ranges, biological responses, and non-inhibitory concentration ranges relevant to the application of nanotechnology in algal bioenergy systems.

Nanoparticle Type	Algal Species	Tested Concentration Range	Reported Biological Response	Suggested Non-Inhibitory Concentration Range	Reference
ZnO nanoparticles	*Chlorella vulgaris*	0.1–10 mg/L	Low concentrations showed limited effects, whereas higher concentrations induced ROS generation, oxidative stress, and growth inhibition	≤1 mg/L	[88]
TiO_2_ nanoparticles	*Chlamydomonas reinhardtii*	0.1–100 mg/L	Growth inhibition increased with nanoparticle concentration due to oxidative stress and light-dependent ROS production	≤10 mg/L	[89]
Ag nanoparticles	*Chlorella vulgaris*	0.01–10 mg/L	Strong toxicity at elevated concentrations associated with Ag^+^ release, ROS accumulation, and reduced photosynthetic activity	≤0.1 mg/L	[90]
Fe_3_O_4_ nanoparticles	*Chlorella vulgaris*	10–500 mg/L	Relatively lower toxicity compared with Ag and ZnO nanoparticles; low concentrations showed minimal effects on growth	≤100 mg/L	[91]
Carbon nanotubes (CNTs)	*Chlorella vulgaris*	5–100 mg/L	Concentration-dependent effects including adsorption onto cell surfaces and reduced growth at higher exposure levels	≤20 mg/L	[92]
Graphene oxide (GO) nanoparticles	*Chlamydomonas reinhardtii*	1–100 mg/L	High concentrations caused oxidative stress, membrane interaction, and reduced photosynthetic performance	≤10 mg/L	[93]

**Table 5:** Recent advances in nanotechnology-enhanced algal bioenergy systems, highlighting key applications of nanomaterials in algal cultivation, lipid enhancement, biofuel conversion, and integrated bioenergy processes.

Focus Area	Nanomaterial/Approach	Key Findings	Reference
Algal biorefinery integration	Metal and hybrid nanoparticles	Increase biomass growth (20–30%), harvesting (80–99%), and conversion efficiency (85–99%)	[96]
Lipid enhancement in microalgae	ZnO nanoparticles	Lipid content increased from ~14% to 48% under controlled NP stress	[97]
Circular nanotechnology in biofuels	Hybrid and carbon-based nanomaterials	Improved catalysis, enzyme immobilization, and sustainability in biofuel cycles	[98]
Enzymatic biodiesel production	Nano-immobilized lipase systems	Enhanced transesterification yield, improved enzyme stability and reusability	[99]
NP–microalgae interaction mechanisms	Metal oxide nanoparticles	Dose-dependent effects: growth stimulation vs. oxidative toxicity in algae	[100]

## Data Availability

No new data were created or analyzed in this study. Data sharing is not applicable to this article.

## Abbreviations

AD	Anaerobic Digestion
BCCU	Biological Carbon Capture and Utilization
C/N	Carbon to Nitrogen Ratio
CA-MFC	Cascade Microbial Fuel Cell
CO_2_	Carbon Dioxide
CNTs	Carbon Nanotubes
COD	Chemical Oxygen Demand
FAME	Fatty Acid Methyl Ester
GHGs	Greenhouse Gases
HTL	Hydrothermal Liquefaction
MFCs	Microbial Fuel Cells
PBR	Photobioreactor
PMFCs	Photosynthetic Microbial Fuel Cells
RSM	Response Surface Methodology
SSF	Simultaneous Saccharification and Fermentation
SAFs	Sustainable aviation fuels
MFSP	Minimum fuel selling price
EROI	Energy return on investment
DIET	Direct interspecies electron transfer
TRL	Technology readiness level
ORR	Oxygen reduction reaction
GO	Graphene oxide
ROS	Reactive oxygen species
TEA	Techno-economic analysis
LCA	Life-cycle assessment

## References

[1] Wang M., Ye X., Bi H., Shen Z. (2024). Microalgae Biofuels: Illuminating the Path to a Sustainable Future amidst Challenges and Opportunities. Biotechnol. Biofuels.

[2] Sharma A., Hussain Mehdi S.E., Shahzad S., Hussain F., Pandey S., Kang W., Oh S.-E. (2026). Microalgal Biorefinery: Innovations in Sustainable Biofuel Production. Green Chem..

[3] Jha S., Singh R., Pandey B.K., Tiwari A.K., Shukla S., Dikshit A., Bhardwaj A.K. (2024). Recent Aspects of Algal Biomass for Sustainable Fuel Production: A Review. Discov. Sustain..

[4] Gaurav K., Neeti K., Singh R. (2024). Microalgae-Based Biodiesel Production and Its Challenges and Future Opportunities: A Review. Green Technol. Sustain..

[5] Wang H., Zheng L., Tan C., Li L., Liu F., Feng H. (2024). Changes in the Microbial Community Structure and Diversity during the Electricity Generation Process of a Microbial Fuel Cell with Algal-Film Cathode. Clean Energy.

[6] Bhadra S., Nayak S., Sevda S. (2024). Simultaneous Organic Wastewater Treatment and Bioelectricity Production in a Dual Chamber Microbial Fuel Cell with Scenedesmus Obliquus Biocathode. Energy Convers. Manag..

[7] Serik A., Kuspanov Z., Daulbayev C. (2026). Cost-Effective Strategies and Technologies for Green Hydrogen Production. Renew. Sustain. Energy Rev..

[8] Park W.G., Kim M., Li S., Kim E., Park E.J., Yoo J., Maile N., Jae J., Kim H., Kim J.R. (2024). A Light-Driven Photosynthetic Microbial Fuel Cell for Carbon-Negative Bioelectricity Production. Sustain. Energy Fuels.

[9] Sarma P.J., Malakar B., Mohanty K. (2024). Self-Sustaining Bioelectricity Generation in Plant-Based Microbial Fuel Cells (PMFCs) with Microalgae-Assisted Oxygen-Reducing Biocathode. Biomass Convers. Bioref..

[10] Piyatilleke S., Thevarajah B., Nimarshana P.H.V., Ariyadasa T.U. (2025). Microalgal Biofuels: Challenges and Prospective in the Framework of Circular Bioeconomy. Energy Nexus.

[11] Bwapwa J.K., Anandraj A., Trois C. (2017). Possibilities for Conversion of Microalgae Oil into Aviation Fuel: A Review. Renew. Sustain. Energy Rev..

[12] Bojarski W., Czekała W., Nowak M., Dach J. (2023). Production of Compost from Logging Residues. Bioresour. Technol..

[13] Tabar Jafar H., Tavakoli O., Nabi Bidhendi G.R., Alizadeh M. (2024). Sustainable Electricity Supply Planning: A Nexus-Based Optimization Approach. Renew. Sustain. Energy Rev..

[14] Chisti Y. (2007). Biodiesel from Microalgae. Biotechnol. Adv..

[15] Devi M.K., Manikandan S., Kumar P.S., Yaashikaa P.R., Oviyapriya M., Rangasamy G. (2023). A Comprehensive Review on Current Trends and Development of Biomethane Production from Food Waste: Circular Economy and Techno Economic Analysis. Fuel.

[16] Ma Y., Li C., Han F., Liu Y., Hani U.E., Zhong Y., Huang D., Chen W., Qian H. (2024). Oral Delivery of Berberine by Liver-Targeted Zwitterionic Nanoparticles to Overcome Multi-Intestinal Barriers and Extend Insulin Treatment Duration. Chem. Eng. J..

[17] Sangtani R., Parida D., Mandal R., Ghosh T., Bala K. (2024). An Untargeted Metabolomic Outlook for Delineating the Reverberations of CO_2_ Exposure to *Scenedesmus* sp.. Algal Res..

[18] Barboza-Rodríguez R., Rodríguez-Jasso R.M., Rosero-Chasoy G., Rosales Aguado M.L., Ruiz H.A. (2024). Photobioreactor Configurations in Cultivating Microalgae Biomass for Biorefinery. Bioresour. Technol..

[19] Penloglou G., Pavlou A., Kiparissides C. (2024). Recent Advancements in Photo-Bioreactors for Microalgae Cultivation: A Brief Overview. Processes.

[20] Scapini T., Woiciechowski A.L., Manzoki M.C., Molina-Aulestia D.T., Martinez-Burgos W.J., Fanka L.S., Duda L.J., Vale A.D.S., De Carvalho J.C., Soccol C.R. (2024). Microalgae-Mediated Biofixation as an Innovative Technology for Flue Gases towards Carbon Neutrality: A Comprehensive Review. J. Environ. Manag..

[21] Krishnamoorthy S., Kuppam C., Mamilla R.C.R. (2024). Evaluation of Carbon Capture Methodologies, Mechanisms, and Improvements for Sustainable Carbon Dioxide Mitigation Using Microalgae. Ind. Biotechnol..

[22] Calatrava V., Gonzalez-Ballester D., Dubini A. (2024). Microalgae for Bioremediation: Advances, Challenges, and Public Perception on Genetic Engineering. BMC Plant Biol..

[23] Kong W., Kong J., Feng S., Yang T., Xu L., Shen B., Bi Y., Lyu H. (2024). Cultivation of Microalgae–Bacteria Consortium by Waste Gas–Waste Water to Achieve CO2 Fixation, Wastewater Purification and Bioproducts Production. Biotechnol. Biofuels.

[24] Lardon L., Hélias A., Sialve B., Steyer J.-P., Bernard O. (2009). Life-Cycle Assessment of Biodiesel Production from Microalgae. Environ. Sci. Technol..

[25] Pittman J.K., Dean A.P., Osundeko O. (2011). The Potential of Sustainable Algal Biofuel Production Using Wastewater Resources. Bioresour. Technol..

[26] Chiu S.-Y., Kao C.-Y., Tsai M.-T., Ong S.-C., Chen C.-H., Lin C.-S. (2009). Lipid Accumulation and CO_2_ Utilization of Nannochloropsis Oculata in Response to CO_2_ Aeration. Bioresour. Technol..

[27] Guo B., Zhang Y., Ha S.-J., Jin Y.-S., Morgenroth E. (2012). Combined Biomimetic and Inorganic Acids Hydrolysis of Hemicellulose in Miscanthus for Bioethanol Production. Bioresour. Technol..

[28] Hu H., Gao K. (2006). Response of Growth and Fatty Acid Compositions of Nannochloropsis Sp. to Environmental Factors Under Elevated CO2 Concentration. Biotechnol. Lett..

[29] Singh J., Dhar D.W. (2019). Overview of Carbon Capture Technology: Microalgal Biorefinery Concept and State-of-the-Art. Front. Mar. Sci..

[30] Kumar K., Dasgupta C.N., Nayak B., Lindblad P., Das D. (2011). Development of Suitable Photobioreactors for CO2 Sequestration Addressing Global Warming Using Green Algae and Cyanobacteria. Bioresour. Technol..

[31] Pandey S., Narayanan I., Selvaraj R., Varadavenkatesan T., Vinayagam R. (2024). Biodiesel Production from Microalgae: A Comprehensive Review on Influential Factors, Transesterification Processes, and Challenges. Fuel.

[32] Rodolfi L., Chini Zittelli G., Bassi N., Padovani G., Biondi N., Bonini G., Tredici M.R. (2009). Microalgae for Oil: Strain Selection, Induction of Lipid Synthesis and Outdoor Mass Cultivation in a Low-cost Photobioreactor. Biotech. Bioeng..

[33] Ponnumsamy V.K., Al-Hazmi H.E., Shobana S., Dharmaraja J., Jadhav D.A., Rajesh Banu J., Piechota G., Igliński B., Kumar V., Bhatnagar A. (2024). A Review on Homogeneous and Heterogeneous Catalytic Microalgal Lipid Extraction and Transesterification for Biofuel Production. Chin. J. Catal..

[34] Kanwal F., Aslam A., Torriero A.A.J. (2025). Microalgae-Based Biodiesel: Integrating AI, CRISPR and Nanotechnology for Sustainable Biofuel Development. Emerg. Top. Life Sci..

[35] Awogbemi O., Ojo A.A., Adeleye S.A. (2024). Advancements in the Application of Metal Oxide Nanocatalysts for Sustainable Biodiesel Production. Discov. Appl. Sci..

[36] Oliva G., Buonerba A., Grassi A., Hasan S.W., Korshin G.V., Zorpas A.A., Belgiorno V., Naddeo V., Zarra T. (2024). Microalgae to Biodiesel: A Novel Green Conversion Method for High-Quality Lipids Recovery and in-Situ Transesterification to Fatty Acid Methyl Esters. J. Environ. Manag..

[37] Biller P., Ross A.B. (2011). Potential Yields and Properties of Oil from the Hydrothermal Liquefaction of Microalgae with Different Biochemical Content. Bioresour. Technol..

[38] Semwal S., Raj T., Kumar R., Christopher J., Gupta R.P., Puri S.K., Kumar R., Ramakumar S.S.V. (2019). Process Optimization and Mass Balance Studies of Pilot Scale Steam Explosion Pretreatment of Rice Straw for Higher Sugar Release. Biomass Bioenergy.

[39] Murray K.E., Shields J.A., Garcia N.D., Healy F.G. (2012). Productivity, Carbon Utilization, and Energy Content of Mass in Scalable Microalgae Systems. Bioresour. Technol..

[40] Ketsub N., Whatmore P., Abbasabadi M., Doherty W.O.S., Kaparaju P., O’Hara I.M., Zhang Z. (2022). Effects of Pretreatment Methods on Biomethane Production Kinetics and Microbial Community by Solid State Anaerobic Digestion of Sugarcane Trash. Bioresour. Technol..

[41] Altun A.F., Kilic M. (2020). Economic Feasibility Analysis with the Parametric Dynamic Simulation of a Single Effect Solar Absorption Cooling System for Various Climatic Regions in Turkey. Renew. Energy.

[42] Malaki M., Jiang X., Wang H., Podila R., Zhang H., Samorì P., Varma R.S. (2023). MXenes: From Past to Future Perspectives. Chem. Eng. J..

[43] Gao H., Zhao R., Wu Z., Ye J., Duan L., Yu R. (2023). New Insights into Exogenous N-Acyl-Homoserine Lactone Manipulation in Biological Nitrogen Removal System against ZnO Nanoparticle Shock. Bioresour. Technol..

[44] Wei J., Sun J., Hou Y., Zhang W., Ren S., Wu W. (2024). Hydroliquefaction of Lignin: Can It Generate Recycle Solvents by Itself?. Fuel.

[45] Vaghela P., Trivedi K., Anand K.G.V., Nayak J., Vyas D., Ghosh A. (2023). Aqueous Homogenate of Fresh Ulva Lactuca for Ameliorating Nutrient Deficiency—A Nutraceutical Alternative to Using Whole Seaweeds. Algal Res..

[46] Li D., Wang F., Zheng X., Zheng Y., Pan X., Li J., Ma X., Yin F., Wang Q. (2025). Lignocellulosic Biomass as Promising Substrate for Polyhydroxyalkanoate Production: Advances and Perspectives. Biotechnol. Adv..

[47] Lim J., Park J., Park K., Kim J. (2024). Design of Hybrid Desalination Process Using Waste Heat and Cold Energy from LNG Power Plant Increasing Energy and Economic Potential. J. Clean. Prod..

[48] Melis A., Zhang L., Forestier M., Ghirardi M.L., Seibert M. (2000). Sustained Photobiological Hydrogen Gas Production upon Reversible Inactivation of Oxygen Evolution in the Green Alga *Chlamydomonas reinhardtii*. Plant Physiol..

[49] Lv Y., Zhou S., Zhang X., Xu Y. (2022). A Smart Self-Balancing Biosystem with Reversible Competitive Adsorption of in-Situ Anion Exchange Resin for Whole-Cell Catalysis Preparation of Lignocellulosic Xylonic Acid. Bioresour. Technol..

[50] Andrade Díaz C., Albers A., Zamora-Ledezma E., Hamelin L. (2024). The Interplay between Bioeconomy and the Maintenance of Long-Term Soil Organic Carbon Stock in Agricultural Soils: A Systematic Review. Renew. Sustain. Energy Rev..

[51] Costa P., Basaglia M., Casella S., Kennes C., Favaro L., Veiga M.C. (2023). Autotrophic Production of Polyhydroxyalkanoates Using Acidogenic-Derived H_2_ and CO_2_ from Fruit Waste. Bioresour. Technol..

[52] Lete A., Raso R., García L., Ruiz J., Arauzo J. (2024). Synthesis of Ketones from Glycerol and 1,2-Propanediol Using Copper and Nickel Catalysts: Unraveling the Impact of Reaction Phase and Active Metal. Fuel.

[53] Elgharbawy A.S., Osman A.I., El Demerdash A.G.M., Sadik W.A., Kasaby M.A., Ali S.E. (2024). Enhancing Biodiesel Production Efficiency with Industrial Waste-Derived Catalysts: Techno-Economic Analysis of Microwave and Ultrasonic Transesterification Methods. Energy Convers. Manag..

[54] Chen X., He T., Yang X., Gan Y., Qing X., Wang J., Huang Y. (2023). Analysis, Environmental Occurrence, Fate and Potential Toxicity of Tire Wear Compounds 6PPD and 6PPD-Quinone. J. Hazard. Mater..

[55] Wang Z., Ma J., Zhu L., He Q., Ke Q., Ke S. (2024). Enhanced Sludge Solubilization by a Fluidized Electrode: Granular Activated Carbon Promoted Electrochemical Oxidation. Bioresour. Technol..

[56] Liu A.S., Li W., Kwok C.Y. (2024). Low Carbon Stabilization of Hong Kong Marine Deposits by Sewage Sludge Ash for Land Reclamation. J. Clean. Prod..

[57] Aditya D.S., Mahadevaprasad K.N., Santhosh K.N., Hemavathi A.B., Halakarni M., Yoon H., Nataraj S.K. (2024). Sustainable and Eco-Friendly Membranes from Sugarcane Bagasse: An Upcycling Approach for Wastewater Treatment and Energy Storage. Chem. Eng. J..

[58] Mussgnug J.H., Klassen V., Schlüter A., Kruse O. (2010). Microalgae as Substrates for Fermentative Biogas Production in a Combined Biorefinery Concept. J. Biotechnol..

[59] Williams P.J.L.B., Laurens L.M.L. (2010). Microalgae as Biodiesel & Biomass Feedstocks: Review & Analysis of the Biochemistry, Energetics & Economics. Energy Environ. Sci..

[60] Park S., Shim J., Song D. (2021). Issues in Calculation of Balance-Point Temperatures for Heating Degree-Days for the Development of Building-Energy Policy. Renew. Sustain. Energy Rev..

[61] Lu L., Li R., Peng T., Fan K., Dai K. (2011). Effects of Rare Earth Ion Modifications on the Photoelectrochemical Properties of ZnO-Based Dye-Sensitized Solar Cells. Renew. Energy.

[62] Ward A.J., Lewis D.M., Green F.B. (2014). Anaerobic Digestion of Algae Biomass: A Review. Algal Res..

[63] Rabaey K., Rozendal R.A. (2010). Microbial Electrosynthesis—Revisiting the Electrical Route for Microbial Production. Nat. Rev. Microbiol..

[64] Rosenbaum M., He Z., Angenent L.T. (2010). Light Energy to Bioelectricity: Photosynthetic Microbial Fuel Cells. Curr. Opin. Biotechnol..

[65] Sharma M., Salama E.-S., Zhang P., Zhang L., Xing X., Yue J., Song Z., Nan L., Yujun S., Li X. (2022). Microalgae-Assisted Microbial Fuel Cells for Electricity Generation Coupled with Wastewater Treatment: Biotechnological Perspective. J. Water Process Eng..

[66] Zieliński M., Rusanowska P., Dudek M., Starowicz A., Barczak Ł., Dębowski M. (2024). Efficiency of Photosynthetic Microbial Fuel Cells (pMFC) Depending on the Type of Microorganisms Inhabiting the Cathode Chamber. Energies.

[67] Qin L., Qin Y., Cui N., Han X., Lu H., Yang T., Liang W. (2024). Photosynthetic Microalgae Microbial Fuel Cells for Bioelectricity Generation and Microalgae Lipid Recovery Using Gd-Co@N-CSs/NF as Cathode. Chem. Eng. J..

[68] Pengadeth D., Prakash Naik S., Sasi A., Mohanakrishna G. (2024). Revisiting the Role of Algal Biocathodes in Microbial Fuel Cells for Bioremediation and Value-Addition. Chem. Eng. J..

[69] Altın N., Uyar B. (2025). Increasing Power Generation and Energy Efficiency with Modified Anodes in Algae-Supported Microbial Fuel Cells. Biomass Conv. Bioref..

[70] Paul V., Agarwal A., Dutt Tripathi A., Sirohi R. (2023). Valorization of Lignin for the Production of Vanillin by Bacillus Aryabhattai NCIM 5503. Bioresour. Technol..

[71] Wang L., Wang H., Huang S., Feng J., Wu Y., Deng J., Liu J., Tu X., Ma X., Li X. (2024). Enhanced Deep Dehalogenation of Brominated DBPs on Vitamin B12 Composite Electrodes by the Synergistic Effect of UV Irradiation: Accelerated Formation of Co-Br Bonds and Enhanced Mediation by Atomic H*. Chem. Eng. J..

[72] Praveen Kumar C.S., Mohan M., Sylas V.P., Thomas A.P., Mechery J., Mohan N. (2026). Role of Microalgae in Fuel Cell Based Wastewater Treatment and Bioelectricity Generation. Discov. Water.

[73] Bhaduri S., Ghosh R., Kumar S., Behera M. (2025). Photosynthetic Microbial Fuel Cells for Wastewater Treatment and Electricity Production. J. Indian Chem. Soc..

[74] Logan B.E., Rabaey K. (2012). Conversion of Wastes into Bioelectricity and Chemicals by Using Microbial Electrochemical Technologies. Science.

[75] Logan B.E., Hamelers B., Rozendal R., Schröder U., Keller J., Freguia S., Aelterman P., Verstraete W., Rabaey K. (2006). Microbial Fuel Cells: Methodology and Technology. Environ. Sci. Technol..

[76] Lv P.-L., Jia C., Wei C.-H., Zhao H.-P., Chen R. (2024). Biochar Modulates Intracellular Electron Transfer for Nitrate Reduction in Denitrifying Anaerobic Methane Oxidizing Archaea. Bioresour. Technol..

[77] Muñoz R., Guieysse B. (2006). Algal–Bacterial Processes for the Treatment of Hazardous Contaminants: A Review. Water Res..

[78] Narayanan M. (2024). Biorefinery Products from Algal Biomass by Advanced Biotechnological and Hydrothermal Liquefaction Approaches. Discov. Appl. Sci..

[79] Malik S., Kishore S., Bora J., Chaudhary V., Kumari A., Kumari P., Kumar L., Bhardwaj A. (2023). A Comprehensive Review on Microalgae-Based Biorefinery as Two-Way Source of Wastewater Treatment and Bioresource Recovery. CLEAN Soil. Air Water.

[80] Silva T.A., Do Couto E.D.A., Assemany P.P., Costa P.A.C., Marques P.A.S.S., Paradela F., Reis A.J.D.D., Calijuri M.L. (2024). Biofuel from Wastewater-Grown Microalgae: A Biorefinery Approach Using Hydrothermal Liquefaction and Catalyst Upgrading. J. Environ. Manag..

[81] Ali H.E.A., El-fayoumy E.A., Soliman R.M., Elkhatat A., Al-Meer S., Elsaid K., Hussein H.A., Zul Helmi Rozaini M., Azmuddin Abdullah M. (2024). Nanoparticle Applications in Algal-Biorefinery for Biofuel Production. Renew. Sustain. Energy Rev..

[82] Yuan X., Gao X., Liu C., Liang W., Xue H., Li Z., Jin H. (2023). Application of Nanomaterials in the Production of Biomolecules in Microalgae: A Review. Mar. Drugs.

[83] Mahmoudi M., Lynch I., Ejtehadi M.R., Monopoli M.P., Bombelli F.B., Laurent S. (2011). Protein−Nanoparticle Interactions: Opportunities and Challenges. Chem. Rev..

[84] Gonzalez-Fernandez C., Sialve B., Molinuevo-Salces B. (2015). Anaerobic Digestion of Microalgal Biomass: Challenges, Opportunities and Research Needs. Bioresour. Technol..

[85] Devarajan Y. (2025). A Comprehensive Review of Advances in Nanoparticle Catalysts for Biodiesel Production. Pet. Sci. Technol..

[86] Wei J., Liang P., Huang X. (2011). Recent Progress in Electrodes for Microbial Fuel Cells. Bioresour. Technol..

[87] Huang L., Lei Y., Zheng P., Chen Q., Wang C., Ma J. (2026). Recent Advances in Mitochondria-Targeted Nano-Drug Delivery Systems for Cancer Therapy. Int. J. Nanomed..

[88] Franklin N.M., Rogers N.J., Apte S.C., Batley G.E., Gadd G.E., Casey P.S. (2007). Comparative Toxicity of Nanoparticulate ZnO, Bulk ZnO, and ZnCl_2_ to a Freshwater Microalga (*Pseudokirchneriella subcapitata*): The Importance of Particle Solubility. Environ. Sci. Technol..

[89] Sundaram T., Rajendran S., Natarajan S., Vinayagam S., Rajamohan R., Lackner M. (2025). Environmental Fate and Transformation of TiO_2_ Nanoparticles: A Comprehensive Assessment. Alex. Eng. J..

[90] Vieira M.C., Torronteras R., Córdoba F., Canalejo A. (2012). Acute Toxicity of Manganese in Goldfish Carassius Auratus Is Associated with Oxidative Stress and Organ Specific Antioxidant Responses. Ecotoxicol. Environ. Saf..

[91] Lei C., Zhang L., Yang K., Zhu L., Lin D. (2016). Toxicity of Iron-Based Nanoparticles to Green Algae: Effects of Particle Size, Crystal Phase, Oxidation State and Environmental Aging. Environ. Pollut..

[92] Schwab F., Bucheli T.D., Lukhele L.P., Magrez A., Nowack B., Sigg L., Knauer K. (2011). Are Carbon Nanotube Effects on Green Algae Caused by Shading and Agglomeration?. Environ. Sci. Technol..

[93] Nogueira P.F.M., Nakabayashi D., Zucolotto V. (2015). The Effects of Graphene Oxide on Green Algae Raphidocelis Subcapitata. Aquat. Toxicol..

[94] Ramesh P., Gupta R., Koventhan C., Muralitharan G., Lo A.-Y., Huang Y.-J., Ramasamy S. (2025). Recent Trends in the Use of Electrode Materials for Microbial Fuel Cells Accentuating the Potential of Photosynthetic Cyanobacteria and Microalgae: A Review. Processes.

[95] Doan L., Pham N.V.H., Nguyen P.B.N., Le Q.N., Phung T.K., Ngo T.T. (2026). Marine Algae as Sustainable Platforms for the Green Synthesis of Metal Nanoparticles. ACS Omega.

[96] Farouk S.M., Tayeb A.M., Abdel-Hamid S.M.S., Osman R.M. (2024). Recent Advances in Transesterification for Sustainable Biodiesel Production, Challenges, and Prospects: A Comprehensive Review. Environ. Sci. Pollut. Res..

[97] Salmeron Covarrubias L.P., Beluri K., Mohammadi Y., Easmin N., Palacios O.A., Sharifan H. (2025). Advanced Nanoenabled Microalgae Systems: Integrating Oxidative Stress-Induced Metabolic Reprogramming and Enhanced Lipid Biosynthesis for Next-Generation Biofuel Production. ACS Appl. Bio Mater..

[98] Lonardi L., Lew-Tong C., Miranda B.D., Viswanaath Shanthamani C., Li E.W., Sahukari B., Poddar H., Satheesh K., Chadha U. (2026). Toward a Circular Nanotechnology for Biofuels: Integrating Sustainable Synthesis, Recovery, and Performance Optimization. J. Mater. Res..

[99] Verma M.L., Dhanya B.S., Wang B., Thakur M., Rani V., Kushwaha R. (2023). Bio-Nanoparticles Mediated Transesterification of Algal Biomass for Biodiesel Production. Sustainability.

[100] Pahariya R., Chauhan A., Ranjan A., Thakur S.K., Tuli H.S., Ramniwas S., Shahwan M., Jindal T. (2024). Impact of Nanoparticles on Microalgae and the Prospects for Biofuel Production: Current Advancements and Future Outlook. J. App Biol. Biotech..

[101] Lau Z.L., Low S.S., Ezeigwe E.R., Chew K.W., Chai W.S., Bhatnagar A., Yap Y.J., Show P.L. (2022). A Review on the Diverse Interactions between Microalgae and Nanomaterials: Growth Variation, Photosynthetic Performance and Toxicity. Bioresour. Technol..

[102] Zheng X., Cooper E., Gillott M., Wood C. (2020). A Practical Review of Alternatives to the Steady Pressurisation Method for Determining Building Airtightness. Renew. Sustain. Energy Rev..

[103] Chatzis A., Orellana E., Gaspari M., Kontogiannopoulos K., Treu L., Zouboulis A., Kougias P.G. (2023). Comparative Study on Packing Materials for Improved Biological Methanation in Trickle Bed Reactors. Bioresour. Technol..

[104] Mandal S., Corcoran A.A. (2022). A Novel Approach to Build Algal Consortia for Sustainable Biomass Production. Algal Res..

[105] Takabe Y., Ida K. (2023). Simultaneous Phosphorus Precipitation and Sludge Thickening by Electrolysis with an Anode Covered by Bivalve Shells. Water Res..

[106] Fois M.G., Van Griensven M., Giselbrecht S., Habibović P., Truckenmüller R.K., Tahmasebi Birgani Z.N. (2024). Mini-Bones: Miniaturized Bone in Vitro Models. Trends Biotechnol..

[107] Ngoma L., Masilela P., Obazu F., Gray V.M. (2011). The Effect of Temperature and Effluent Recycle Rate on Hydrogen Production by Undefined Bacterial Granules. Bioresour. Technol..

[108] Davis R.E., Fishman D.B., Frank E.D., Johnson M.C., Jones S.B., Kinchin C.M., Skaggs R.L., Venteris E.R., Wigmosta M.S. (2014). Integrated Evaluation of Cost, Emissions, and Resource Potential for Algal Biofuels at the National Scale. Environ. Sci. Technol..

[109] Clarens A.F., Resurreccion E.P., White M.A., Colosi L.M. (2010). Environmental Life Cycle Comparison of Algae to Other Bioenergy Feedstocks. Environ. Sci. Technol..

[110] Hallenbeck P.C., Ghosh D. (2009). Advances in Fermentative Biohydrogen Production: The Way Forward?. Trends Biotechnol..

[111] Lubitz W., Ogata H., Rüdiger O., Reijerse E. (2014). Hydrogenases. Chem. Rev..

[112] Brahim T., Jemni A. (2026). Green Hydrogen Production: A Review of Technologies, Challenges, and Hybrid System Optimization. Renew. Sustain. Energy Rev..

